# Gene fusions, micro-exons and splice variants define stress signaling by AP2/ERF and WRKY transcription factors in the sesame pan-genome

**DOI:** 10.3389/fpls.2022.1076229

**Published:** 2022-12-22

**Authors:** Ramya Parakkunnel, Bhojaraja Naik K, Girimalla Vanishree, Susmita C, Supriya Purru, Udaya Bhaskar K, KV. Bhat, Sanjay Kumar

**Affiliations:** ^1^ ICAR- Indian Institute of Seed Science, Regional Station, Gandhi Krishi Vigyana Kendra (GKVK) Campus, Bengaluru, India; ^2^ ICAR- Indian Institute of Seed Science, Mau, Uttar Pradesh, India; ^3^ ICAR- National Academy of Agricultural Research Management, Hyderabad, Telengana, India; ^4^ Division of Genomic Resources, ICAR- National Bureau of Plant Genetic Resources, New Delhi, India

**Keywords:** AP2/ERF, defense, duplication, evolution, gene fusion, micro-exon, sesame, WRKY

## Abstract

Evolutionary dynamics of AP2/ERF and WRKY genes, the major components of defense response were studied extensively in the sesame pan-genome. Massive variation was observed for gene copy numbers, genome location, domain structure, exon-intron structure and protein parameters. In the pan-genome, 63% of AP2/ERF members were devoid of introns whereas >99% of WRKY genes contained multiple introns. AP2 subfamily was found to be micro-exon rich with the adjoining intronic sequences sharing sequence similarity to many stress-responsive and fatty acid metabolism genes. WRKY family included extensive multi-domain gene fusions where the additional domains significantly enhanced gene and exonic sizes as well as gene copy numbers. The fusion genes were found to have roles in acquired immunity, stress response, cell and membrane integrity as well as ROS signaling. The individual genomes shared extensive synteny and collinearity although ecological adaptation was evident among the Chinese and Indian accessions. Significant positive selection effects were noticed for both micro-exon and multi-domain genes. Splice variants with changes in acceptor, donor and branch sites were common and 6-7 splice variants were detected per gene. The study ascertained vital roles of lipid metabolism and chlorophyll biosynthesis in the defense response and stress signaling pathways. 60% of the studied genes localized in the nucleus while 20% preferred chloroplast. Unique cis-element distribution was noticed in the upstream promoter region with MYB and STRE in WRKY genes while MYC was present in the AP2/ERF genes. Intron-less genes exhibited great diversity in the promoter sequences wherein the predominance of dosage effect indicated variable gene expression levels. Mimicking the NBS-LRR genes, a chloroplast localized WRKY gene, Swetha_24868, with additional domains of chorismate mutase, cAMP and voltage-dependent potassium channel was found to act as a master regulator of defense signaling, triggering immunity and reducing ROS levels.

## 1 Introduction

Transcription Factors (TF) are an important class of genes involved in the regulation of plant response under many biotic and abiotic stress conditions. APETALA2/ETHYLENE RESPONSIVE FACTOR (AP2/ERF) and WRKY genes are major components of complex regulatory networks in plants during developmental processes and defense responses (Abdullah-Zawawi et al., 2021; Li et al., 2021). The AP2/ERF transcription factors contain a conserved AP2/ERF domain of about 60 to 70 amino acids, and consist of five subfamilies, AP2, ERF, DREB (Dehydration Responsive Element-Binding), RAV (Related to ABI3/VP1) and Soloist based on the number of AP2/ERF domains and the presence of other DNA binding domains (Dossa et al., 2016). The differential expression of AP2/ERF genes under multiple stresses of heat, drought, cold and salinity has been characterized in wheat (Riaz et al., 2021), *Brassica napus* (Ghorbani et al., 2020), pear (Li et al., 2018) and sesame (Dossa et al., 2016) while secondary metabolite biosynthesis was studied in eggplant (Li et al., 2021). WRKY TF family is the seventh largest and contains the signature domain of 60-70 amino acids representing WRKYGQK/WRKYGKK at the N-terminus and a Zn-finger domain at the C-terminus (Yang et al., 2017). Genome-wide characterization of WRKY genes has been reported in soybean (Yang et al., 2017) against cyst nematode, sorghum (Baillo et al., 2020) against multiple stress responses, the biotic and abiotic stress response in sunflower (Liu et al., 2020), the abiotic stress response in apple (Qin et al., 2022) and sesame (Li et al., 2017).

Sesame, (*Sesamum indicum* L.) belonging to the family Pedaliaceae is an ancient oilseed crop cultivated in the tropical and sub-tropical regions of the world by poor and marginal farmers. Majority of the wild species of the genus *Sesamum* are native to sub-Saharan Africa however, domestication happened in India (Bedigian, 2003). Recently, a sesame pan-genome assembly of 554.05Mb comprising modern cultivars and landraces was developed including 26472 orthologous gene clusters (Yu et al., 2019). In order to exploit the full potential of genetic diversity present in the germplasm of the crop plants, trait-based investigations in the different cultivars of the same crop, adapted to widely different agro ecological conditions are imperative. In this context, the pan-genome offers a viable alternative presenting researchers with useful genetic variation in a number of component genomes as against a single reference genome. Particularly in crops like sesame where domestication syndrome is evident in the genome for many useful traits, the constructed pan-genomes become a valuable tool facilitating researchers in mining natural variation for molecular breeding (Yu et al., 2019). In addition, the fine dissection of homologs and paralogs at exonic, intronic and promoter sequence levels attune evolutionary studies with limitless possibilities. In the present study, accelerated evolution under multiple stress conditions is discussed in cultivars adapted to wider climatic niches and parts of the sesame pan-genome. The homologs for AP2/ERF and WRKY genes are studied in relation to evolutionary adaptations, gene duplications, gene fusions, variations in cis-element architecture and variations in splicing machinery involved in defense response and development.

## 2 Materials and methods

### 2.1 Identification of AP2/ERF and WRKY genes from the sesame pan-genome

The pan-genome assembly include *S. indicum* var Zhongzhi-13, *S. indicum* var Yuzhi-11, *S. indicum* var Baizhima, *S. indicum* var Mishouzhima (all from China) and the Indian variety *S. indicum* var Swetha. Here after the component genomes will be referred to as Zhongzhi-13, Yuzhi-11, Baizhima, Mishouzhima and Swetha respectively. From the sesame pan-genome (Yu et al., 2019); the CDS, protein and gff files were used to identify sequences corresponding to Pfam ids PF00847 and PF03106 representing AP2/ERF and WRKY genes. The ‘gff’ files were processed with excel and exon-intron size was identified. The genes were mapped onto the chromosome using ‘gene location visualize’ tool from TB tools (Chen et al., 2020). The exon/intron structures were determined by the gene structure display server (Hu et al., 2015), (http://gsds.cbi.pku.edu.cn/). The different domains were categorized by SMART (Letunic and Bork, 2018) tool (http://smart.embl-heidelberg.de/). The conserved domains in the sesame CDS were identified using NCBI-CDD database search tool (Marchler-Bauer et al., 2017). Protein parameters were worked out using ‘ProtParam’ tool (https://web.expasy.org/protparam/). The exon, intron, and micro-exon distribution for AP2/ERF and WRKY genes from sesame pan-genome was visualized with an online version of CIRCOS available at (http://mkweb.bcgsc.ca/tableviewer/). The package ‘ggplot2’ was used for the visualization of all other data in R.

### 2.2 Phylogenetic analysis

The initial phylogenetic analysis of Swetha protein sequences, Arabidopsis and rice was carried out through NJ algorithm in MEGA X (Kumar et al., 2018b) using the Jones-Taylor-Thornton distance matrix with 500 bootstrap replications. Multiple sequence alignment was done using CLUSTAL X ver. 2.1. Arabidopsis and rice homologs were identified from The Arabidopsis Information Resource (TAIR) available at https://www.arabidopsis.org/ and Plant Transcription Factor Data Base (PlantTFDB ver.5.0) available at http://planttfdb.gao-lab.org/ respectively. The reported classification of Arabidopsis and rice was used for classifying Swetha AP2/ERF and WRKY genes. This classification was further extended to the sesame pan-genome.

The pan-genome protein sequences were aligned using CLUSTAL X ver. 2.1 and were subjected to Bayesian phylogenetic inference using MCMC by BEAST ver. 2.6.6 (Bouckaert et al., 2019). The input ‘XML’ files were generated using BEAUti interface (Drummond et al., 2012) with the model ‘GTR+I+G’ and the ‘Yule speciation process’ under a strict clock model. Two independent runs of 10000000 generations of MCMC chains were produced and sampled after every 5000 generations. TRACER ver1.7.1 (Rambaut et al., 2018) was used for combining the files and the plotted posterior estimates were inspected. The first 10,000 trees were discarded as burn-in, and the rest of the samples were summarized in a maximum clade credibility tree using TreeAnnotator ver. 2.6.6 with a posterior probability limit of 0.5. Means and 95% higher posterior densities (HPDs) obtained from the combined output of TRACER were used for the construction of trees using FigTree ver.1.4.4 (http://tree.bio.ed.ac.uk/software/figtree/) with median heights.

### 2.3 Synteny and collinearity

Based on phylogeny genes were ordered as exon-intron sequences and the micro-exon sequences were identified. The 200bp upstream and downstream region including the micro-exon was analyzed for the presence of protein-coding domains using BLASTX search. The exon-intron size was estimated in excel. Based on BEAST phylogeny, gene pair files were created and used to calculate the non-synonymous/synonymous (Ka/Ks) mutation ratio with TB tools from the respective CDS, protein and gff data (Chen et al., 2020). *Arabidopsis thaliana* and *Oryza sativa* ssp *indica* genomes were downloaded from the Phytozome (https://phytozome-next.jgi.doe.gov/). The syntenic relationships between sesame, Arabidopsis and rice genomes were probed with MCScanX using TB tools. Based on the results of MCScanX sesame genes were classified as WGD or segmental duplicates. The evolutionary time in million years ago (MYA) for each orthologous pair was calculated using the formula, T= Ks/2r (Moghaddam et al., 2021); where ‘r’ the rate of mutation was kept as 1.5x10^-9^ based on the age of divergence of Zhongzhi-13 and Swetha genomes (Yu et al., 2019).

### 2.4 Alternative splicing and protein-protein interaction

The multi-exon homologs were probed for intron-exon size variation and such pairs were selected for identification of splice site and SRP protein site mutations (Kharabian, 2010; Karlik, 2021) through ESEfinder2.0 (http://krainer01.cshl.edu/tools/ESE2/). The cut-off for splice donor and splice acceptor sites was kept at 6.9 and for branch site was 2.0. The splice SRP protein sequences (SF1 & SF2) of ESE finder was used as a rough guideline for prediction and the identified sites were compared among the component genomes for probable mutations. The deviation in position and score of splice sites and SRP proteins were noted for protein homologs and the splicing mechanism was devised based on a comparison with the exon-intron data. The conserved motifs in the sesame proteins were identified using the MEME program (https://meme-suite.org/meme/tools/meme) using parameters: maximum number of motifs = 10; optimum width of motifs = 15–50. The identified motifs were subjected to ‘GoMo’ scan to identify ‘GO’ terms associated with the biological function (http://meme-suite.org/tools/gomo). Protein-protein interaction network was visualized with the help of STRING ver. 11.5 (https://string-db.org/) and plotted with the help of Cytoscape ver. 3.9.1. Prediction of protein sub-cellular localization was done with the help of WoLF PSORT tool (https://wolfpsort.hgc.jp/). The cis-element identification was done by subjecting upstream 2000bp from the start codon of selected AP2/ERF and WRKY sequences from Swetha and Zhongzhi13 genomes to PLANT CARE (https://bioinformatics.psb.ugent.be/webtools/plantcare/html/) and comparing with the reported Arabidopsis cis-elements.

### 2.5 Expression profiles of AP2/ERF and WRKY genes

Microarray data of AP2/ERF and WRKY genes were obtained from NCBI-Gene Expression Omnibus (GEO) database under the accession numbers GSE81039, GSE102714, GSE81325, GSE49418, GSE55835 and GSE167174. The data were properly grouped as per study objectives and was analyzed through GEO2R. After processing the transcriptome data, heat maps were constructed in R using the adjusted P-values for AP2/ERF and WRKY genes having significant logFC or F-statistics (more than two groups defined) for each accession.

## 3 Results

### 3.1 Identification of AP2/ERF and WRKY genes from sesame pan-genome

A total of 704 AP2/ERF genes and 387 WRKY genes were identified in the sesame pan-genome (**Table 1**). The lowest number of AP2/ERF genes was observed in the Yuzhi-11 genome (131) whereas the genomes of the Chinese landrace ‘Mishouzhima’ and the Indian variety ‘Swetha’ contained 145 each. As for WRKY genes, the Chinese cultivar (Yuzhi-11) and the landrace (Baizhima) contained 73 genes each whereas Swetha contained 89 genes.

**Table 1:** Summary statistics of identified AP2/ERF and WRKY genes in sesame pan-genome.

Genome	Genes	Intron	Exon	Micro_Ex	Exon/Gene	Exon/Intron	Micro_ex/Gene	Micro_ex/Exon	Intron/Gene
AP2/ERF									
Baizhima	144	210	354	41	2.458333	1.685714	0.284722	0.115819	1.458333
Mishouzhima	145	208	353	34	2.434483	1.697115	0.234483	0.096317	1.434483
Swetha	145	356	501	51	3.455172	1.407303	0.351724	0.101796	2.455172
Yuzhi11	131	196	327	32	2.496183	1.668367	0.244275	0.097859	1.496183
Zhongzhi13	139	213	352	37	2.532374	1.652582	0.266187	0.105114	1.532374
Total	704	1183	1887	195	2.680398	1.595097	0.276989	0.103339	1.680398
WRKY									
Baizhima	73	221	294	4	4.027397	1.330317	0.054795	0.013605	3.027397
Mishouzhima	76	231	307	6	4.039474	1.329004	0.078947	0.019544	3.039474
Swetha	89	355	444	12	4.988764	1.250704	0.134831	0.027027	3.988764
Yuzhi11	73	216	289	4	3.958904	1.337963	0.054795	0.013841	2.958904
Zhongzhi13	76	221	297	6	3.907895	1.343891	0.078947	0.020202	2.907895
Total	387	1244	1631	32	4.21447	1.311093	0.082687	0.01962	3.21447

The numbers of genes, exons, introns and micro_exons are given for both the families for all the five component genomes.

#### 3.1.1 Chromosomal location of AP2 and WRKY genes

**Figure 1:**
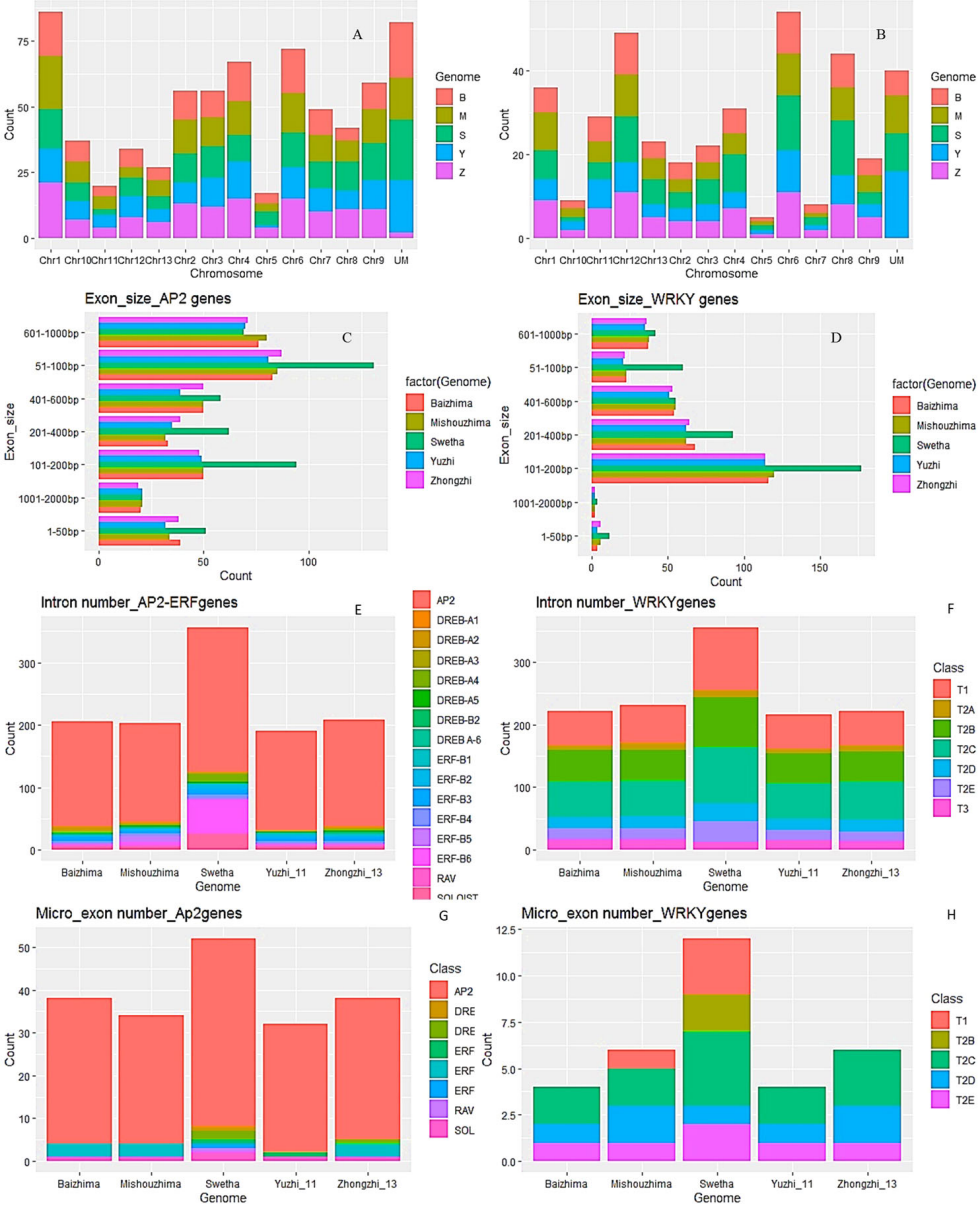
Salient features of AP2/ERF and WRKY genes in the sesame pan-genome. Chromosomal distribution **(A, B)** while exon size of AP2/ERF and WRKY genes in individual genomes **(C–F)** represent intron numbers; **(G, H)** represent micro-exon numbers of AP2/ERF and WRKY genes in individual genomes as per individual genome, subfamily and class wise.

The AP2/ERF and WRKY genes were distributed all over the 13 chromosomes with variations in individual genomes. Maximum AP2/ERF genes were located on chr-1 and chr-6 while the least numbers were observed on chr-5 and chr-11. For WRKY genes, chr-6 had the highest number including 10-13 genes from individual genomes whereas chr-5 contained a single gene in all the genomes. Moreover, 82 AP2/ERF and 39 WRKY genes were not mapped to any chromosome. Details are in **Figure 1A, B**), **SI-1A , SI-2A** while **SI-18** gives the chromosomal location of mapped genes.

### 3.2 Phylogenetic analysis of AP2/ERF and WRKY genes

**Figure 2:**
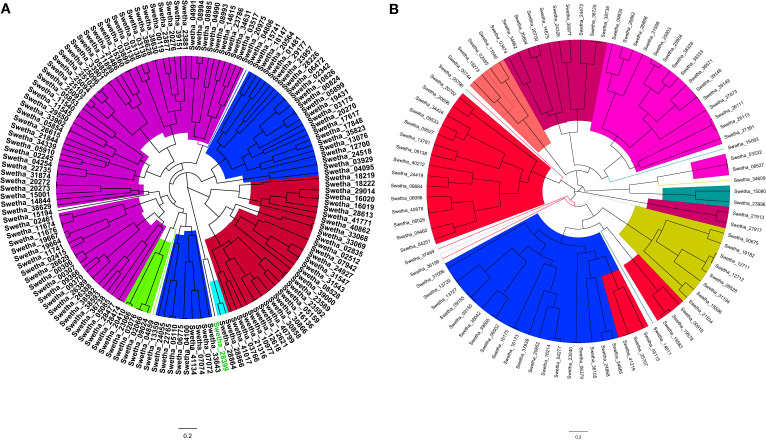
The MCMC phylogeny tree of **(A)** AP2/ERF and **(B)** WRKY families in the Swetha genome based on Arabidopsis and rice classification. In **(A)** the colour codes are as follows: Blue=DREB; Magenta=ERF; Red=AP2; Green=RAV and Cyan=SOLOIST. In **(B)** the different subfamilies are as follows; T1=Blue; T2A= Orange; T2B=Magenta, T2C=Red; T2D=Lime yellow; T2E= Purple and T3= Olive.

The phylogenetic analysis of AP2/ERF and WRKY genes of sesame was conducted using the multiple sequence alignment results of ‘Swetha’ protein sequences along with Arabidopsis homologs. Bayesian phylogeny trees were constructed for each gene family and the individual members were classified based on already published Arabidopsis gene classification. Afterward, the newly defined classifications of Swetha proteins were extended to the whole of the sesame pan-genome. The 145 AP2/ERF genes identified in Swetha genome were further classified as belonging to different subfamilies of DREB, ERF, and AP2. The ERF subfamily had the maximum share (70), followed by DREB (32), AP2 (31), RAV (9) and Soloist (3). The ERF family was further classified as different groups B1-B6 and contained 16, 7, 22, 7, 5 and13 genes respectively. The DREB subfamily contained 5 groups A1, A2, A4, A5 and A6 including 3, 4, 13, 9 and 4 members respectively. The WRKY genes were also classified as belonging to subclasses T1, T2 and T3 based on the number of WRKY domains and the type of zinc-finger motif present. Among the 89 genes present in Swetha T2 had the highest share (66), followed by T1 (19) while T3 contained only 4 genes. The details of gene classification in ‘Swetha’ are given in **Figures 2A, B**), **SI-1B, SI-2B**.

The ERF, AP2, RAV and Soloist domains retained similarity all through the pan-genome with frequent domain changes noticed in closely related clusters of proteins. The ERF-B3 domain exhibited sequence similarity to ERF-B1, AP2, Soloist, DREB-A5 and DREB-A4 domain proteins. The A4 domain genes showed sequence similarity to ERF-B4 and ERF-B1 genes along with AP2 genes. DREB-A2 domain genes in turn were found to be related to ERF-B6 and ERF-B1 domain genes. Among the DREB subfamily, A1 was more conserved where group-specific clustering was observed. The frequent domain changes, segmental duplication and exonic changes made the phylogeny reconstruction quite tedious in AP2/ERF family. However, for WRKY genes sequence conservation was noticed all through the pan-genome. Among the 387 WRKY genes identified the T1, T2 and T3 groups had 80, 285 and 22 genes respectively. T3 genes in the pan-genome shared sequence similarity with T2D and T2A whereas Swetha T3 genes were more related to T2D and T2E groups. One set of T3 genes was found solely in the Chinese accessions. T1 genes of the pan-genome were found more related to T2E genes whereas the Swetha genes were closer to the T2C genes. Details are given as SI-19 and SI- 20.

### 3.3 Gene structure of homologous genes in pan-genome

Based on multiple sequence alignment and phylogeny, the homologs were identified for AP2/ERF and WRKY genes from individual genomes. The exon-intron structure and sizes of exons and introns were identified for each homologous set (SI-4 and SI-6).

#### 3.3.1 Intron number and size variants

In the AP2/ERF gene family, 443 genes were found to be devoid of introns (**Figure 1E**). Mishouzhima (99) had the largest while Swetha (77) had the least number of intron-less genes. 68 genes of Swetha contained introns while for Mishouzhima and Yuzhi genomes, only 47 genes had introns. 36 introns were present in the gene Swetha_28474, whereas Swetha_02835 had 20 introns. The mRNAs of these genes spanned 30 kb and 17kb respectively. In the AP2/ERF family, 92 genes had single intron, 36 genes had 7 introns, 32 genes had 5 introns while 3 and 4 intron genes were less frequent. The intron size varied from 34bp in Mishuozhima_01646 (SI-4) located in chromosome-1 to 43.6kb in Swetha_11741 in chromosome-3 (SI-4). Two other genes (Swetha_11742 and Swetha_11743) were found nested in this huge intron coding for Alpha-amylase inhibitor and phospholipase D respectively. Another intron of size 28kb was observed in Yuzhi11 _12343 in chromosome-8. However, this large intronic region did not harbor any additional genes.

In the WRKY family, except for T2C genes, Swetha_15083 and Baizhima_02279; all the others had introns (**Figure 1F**). The largest number of introns noticed in a single gene was 17 in Swetha_24868 belonging to the T1 group while Swetha_09533 had 14 introns. All the genomes shared a common gene with 11 introns belonging to T2C. The smallest intron noticed was of size 31bp and was present in Zhongzhi13_04758 and its homologs in Baizhima, Mishouzhima and Yuzhi11. This gene present in Chinese accessions is worth noticing for its sequence conservation and exonic as well as intronic number and size conservation. The largest intron noticed was 35kb in the gene Swetha_09138 in chromosome-2 and the mRNA spanned 38kb in length. This huge intronic region was found to harbor two additional genes namely Swetha_09139 and Swetha_09140, coding for AB hydrolase1 and pentatricopeptide repeat-containing protein respectively. Another Swetha gene, Swetha_02485 in chromosome-1 also harbored a huge intron of size ~19kb. A Methyladenine glycosylase gene (Swetha_02486) was found nested in this intronic region. 153 WRKY genes from the sesame pan-genome contained 2 introns, while 79 had 4 and 72 had 3 introns. The variation in intron numbers in the individual genomes is represented in (SI-1B, SI-1C, SI-2B and SI-2C).

#### 3.3.2 Exon number and size variants

The individual genomes differed greatly in exon number and size. In the AP2/ERF family, the total number of exons detected varied widely although gene number was comparable. Swetha genome contained a large number of multi-exon genes with total exons of 486 against 351 and 352 in Baizhima and Mishouzhima respectively. The number of exons in Zhongzhi-13 is 373 whereas 327 exons were found in Yuzhi-11. 51-100bp exons were most common while exons of size >1kb were least common. In the Swetha genome, 101-200bp exons were the second most common against 601-1000bp exons in all the Chinese accessions. The smallest exon noticed was of 3bp present in all the genomes while the largest exon was 1262bp in Yuzhi11 genome.

In the WRKY family, 443 exons were detected in Swetha against 297 in Zhongzhi-13. 101-200bp size exons were most common in all the genomes followed by 201-400bp and 401-600bp exons. The smallest exon detected was of 3bp (Swetha_06086) in Swetha genome, whereas among WRKY genes from the Chinese accessions the 3 bp exon was observed only in Mishuozhima_16023. The largest exon detected was of 1287bp, present as a single exon gene conserved in the pan-genome. Details of exon number and size distribution are given in **Figure 1C, D**, **Table 1**, **SI-1C, 1D**. In addition, a number of gene duplication events were found unique among WRKY genes in the Swetha genome resulting in increased gene copy numbers. The duplicated gene was found positioned in the same chromosome with a different location or in a different chromosome. These genes differed in intron number (Swetha_20694 and Swetha_20700), exon size (Swetha_21913 and Swetha_21917; Swetha_03532 and Swetha_06527), conversion of exon into micro-exon (Swetha_38738 and Swetha_38725), transposon induced insertion or deletion (Swetha_21534, Swetha_18596 and Swetha_00675) to name a few. Although such duplicates occurred in the AP2-ERF family also, the genome-wise distribution was more or less equal. The details are given in SI-1A, SI-2A, SI-4, 5, 6, 7 and 8.

#### 3.3.3 Micro-exons in sesame pan-genome

The exonic fragments of length <51bp were classified as micro-exons (Song et al., 2020) and we found 227 micro-exons in the sesame pan-genome varying in size from 3bp to 50 bp (SI-3A, SI-3B, SI-4 and SI-6). AP2/ERF gene family had 195 micro-exons distributed along 133 genes with Swetha contributing a major share of 36. Baizhima had 26 micro-exon genes whereas the numbers in Mishouzhima, Yuzhi11 and Zhongzhi-13 were 25, 21 and 25 respectively. Micro-exon genes were present in all subfamilies in Swetha genome Swetha had a total of 51 micro-exons with multiple micro-exons noticed in many genes. The gene Swetha_18222 had 5 micro exons out of the total 11 exons and had the largest micro exon count for a single gene. The 6^th^ exon was the most preferred position for micro-exons whereas after the 10^th^ exon the presence of micro-exons becomes very rare. Among the micro-exon containing genes, 38 genes (6 sets) including duplicates were found to have exonic and intronic sequence conservation across the pan-genome while 7 sets (28 genes) were found to have sequence conservation across 4 genomes. 8 sets (40 genes) were found to have exonic divergence while retaining the micro-exon conservancy with the change noted particularly in the 1^st^ or the last exon. The bulk of micro-exons (171) were contributed by the AP2 subfamily while presence was noticed in DREB-A2, DREB-A4, ERF-B1, ERF-B3, ERF-B6, RAV and SOLOIST families. 32 micro-exons were noticed in the WRKY family with a major share (12) contributed by Swetha. These were distributed into T1 (2 genes), T2B (1 gene with 2 micro-exons), T2C (5 genes), T2D (1 gene) and T2E (2 genes). The T2 WRKY genes of Chinese accessions contained 19 micro-exons while class T1 had a single micro-exon. Details are in **Figure 1G, H**), **SI-3A, SI-3B**. The micro-exonic region and the adjoining intronic sequences were found to share sequence similarity to many functional domains and genes such as glycosyltransferase, phospholipase (LCAT3), pectate lyase, ribonuclease3, ASGR-BBM like2, asparagine synthase(common in all the genomes), chromatin modification-related protein EAF-1, G-protein coupled receptor1, PAS domain S-box containing protein, TonB dependent receptor, transmembrane helix (common), aquaporin, integrase as well as transposon ‘Tpn104’. Details are given in **Table 2** while **SI-8** represents transposon distribution in coding sequences.

**Table 2:** The functional domains identified in the 200bp upstream and downstream region including the micro-exon in the sesame pan-genome. The reported functions and the references are also given.

Sl. No	Domain name	Function	Reference
1	glycosyl transferase	1) glucosylation of lignans in sesame seed 2) maintenance of cell membrane integrity during abiotic stress	Ono et al., 2020; Shi et al., 2020
2	lecithin: cholesterol acyltransferase (LCAT3)	1) Lipid metabolism and production of specialized fatty acids 2) Defense response against *Podosphaera xanthii* in cucumber	Xu et al., 2020; Ming et al., 2022
3	Pectate lyase	1) pectin degradation 2) plant defense response and apoptopsis 3) ROS accumulation	Uluisik and Seymour, 2020; Chen et al., 2021; He et al., 2021
4	Ribonuclease3	1)RNA maturation, modification and splicing 2) antiviral defense	Olmedo and Guzmán, 2008; Aguado and tenOever, 2018
5	ASGR-BBM like	1)Apomixis	Worthington et al., 2019
6	asparagine synthase	1)Multiple nutrient stress response 2) drought and nutritional stress in wheat	Curtis et al., 2018
7	EAF-1	1)Important component of chromatin remodeling complex NuA4 in Arabidopsis 2) Regulation of plant stress response	Wang et al., 2019;
8	G-protein coupled receptor1	1)Multiple abiotic stresses like salinity, drought, extreme temperature and high light intensity	Wu and Urano, 2018
9	PAS domain S-box containing protein	1) Circardian clock 2) Ecological adaptation to diverse stress stimulii	Vogt and Schippers, 2015; Tischkau, 2020.
10	TonB receptor	1)Metal resistance	Theriault and Nkongolo, 2017
11	Aquaporin	1)Stress tolerance and seed germination 2)Plant-pathogen interaction	Liu et al., 2013
12	Integrase	1) Retrotransposon introgression	Suh, 2021

### 3.4 Protein diversity of AP2/ERF and WRKY homologs

**Figure 3:**
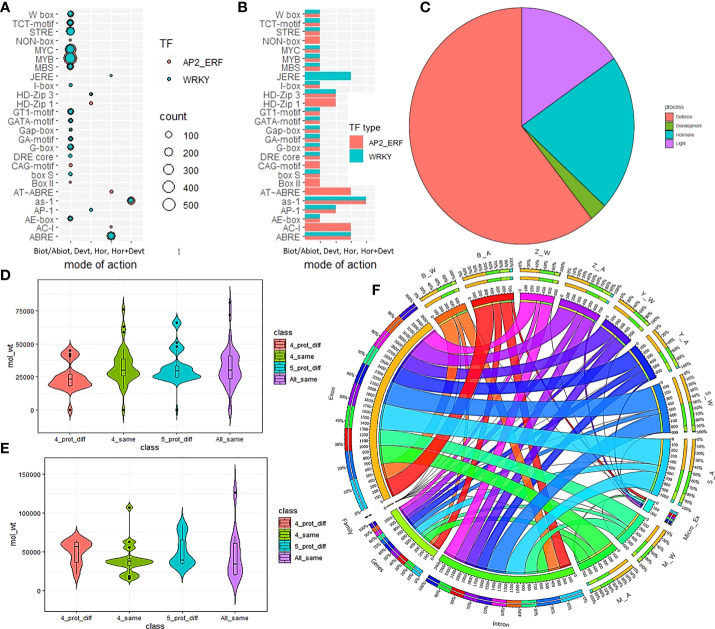
Distribution of cis-elements and protein parameters in the sesame pan-genome for AP2/ERF and WRKY genes. **(A, B)** represent the cis-element distribution and mode of action in the major categories of biotic/abiotic stress response, development, hormone as well as hormone and development. The pie chart **(C)** indicates the number of promoters involved in different functions. The difference in protein parameters in AP2/ERF and WRKY genes are given in **(D, E)** while **(F)** gives the CIRCOS plot for pan-genome.

The shortest protein observed in the AP2/ERF family was of length105AA and belonged to the ERF sub-family gene, Swetha_03899 which also had the lowest molecular weight (11.8kDa). The largest protein among AP2/ERF family also belonged to another ERF gene, Swetha_28474 with1980 AA and a molecular weight of 220.5kDa. Among the AP2 sub-family, Swetha_02835 measured a length and weight of 1937AA and 217.2kDa respectively. A lot of protein variants were found among homologs, where although the protein length was conserved; AA mutations in the functional domains gave way to altered protein parameters. Based on the occurrence of similar length homologs in all or at least 4 genomes the proteins were classified as all same (5 genomes), all different, 4 same and 4 different. We found 36 genes to have all the parameters conserved across all the 5 gnomes whereas conservation in 4 genomes was noticed for 29 genes. Even with the same protein length, significant variation was observed in the molecular weight and iso-electric point and was categorized as differing for all 5 cases (17 genes) or all 4 cases (13 genes). Among WRKY genes, the shortest protein was of length 129AA and was present in all the Chinese accessions homologous to Zhongzhi13_22905 and belonged to the T1 group. Another T1 gene, Swetha_24868 recorded the largest and heaviest WRKY protein with a length and weight of 1261AA and 142kb respectively. The second largest protein was common in all five genomes and belonged to T2C with 1141 AA and 11 introns, homologous to Zhongzhi13_28134. In the WRKY subfamily, the Chinese accessions showed a greater conservation pattern with regard to length and other protein parameters. We found 10 genes to have all the protein parameters conserved in all 5 genomes whereas 4 similar homologs were found in 22 genes majorly including Chinese accessions. Details in **Figure 3D, E**, **SI-1A, SI-2A**. The diversity of pan-genome is represented as circos plot (**Figure 3F**).

### 3.5 Multi-domain genes in sesame pan-genome

**Figure 4:**
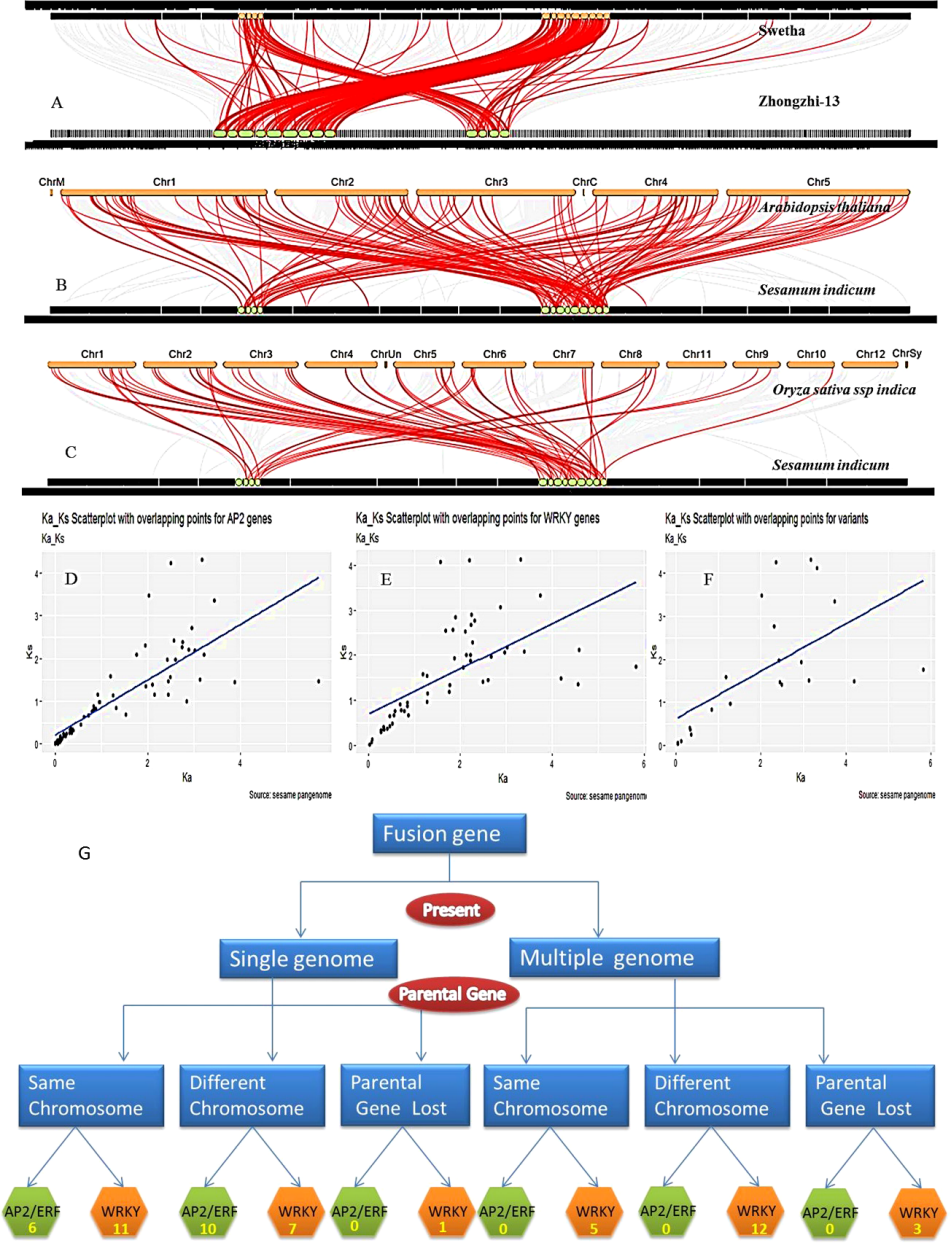
Synteny, collinearity and origin of gene fusions in pan-genome. **(A)** Synteny between Swetha and Zhongzhi genomes. **(B)** Sesame and Arabidopsis genomes **(C)** Sesame and rice genomes **(D–F)** specify selection pressure in AP2/ERF, WRKY and Swetha duplicates respectively. **(G)** Represent the origin of fusion genes in sesame pan-genome.

In addition to the main AP2/ERF and WRKY domains, we found additional domains in 55 genes in the sesame pan-genome possibly as a result of gene fusion. WRKY family had 39 multi-domain genes whereas AP2/ERF had 16 such genes. Maximum cases of multi-domain genes were noticed in the Swetha genome including 20 WRKY and 12 AP2/ERF genes. The multi-domain genes found in genomes of Baizhima, Mishouzhima, Yuzhi11 and Zhongzhi-13 were 6, 6, 6 and 4 respectively. Details are given in **Table 3** and **Figure 3F**. Among the WRKY genes, 4 sets of multi-domain genes were common and were present in all the genomes. In addition to the WRKY domain, these contained additional domains like Arginine/lysine/ornithine decarboxylase, ATP-dependent metalloprotease FtsH, eukaryotic translation initiation factor 5A, DUF3084 and Lung-7-transmembrane receptor. Moreover end to end fusion of genes resulting in multiple functional AP2/ERF and WRKY domains was also common and up to 4 copies of the active domain were noticed for both the families. Other common domains associated were mostly enzymes like kinases, reverse transcriptases, hydrolases, peroxidases, carboxylases, methyl transferases, etc. The inclusion of additional domains resulted in larger-sized genomes with added exons which completely altered gene structure and splicing mechanism. Fusion genes were noticed as novel genes in a single genome or were present in multiple genomes. Based on the location of parental and fusion genes in the genome a detailed classification was made and given in **Figure 4G**.

**Table 3:** Multi-domain genes identified in the sesame pan-genome evolved through gene fusion.

Gene	Introns	Domains	Reported functions	Reference
Swetha_00824	4	AP2+exostosin	Defense, Salt stress, endomembrane organisation	Li et al., 2013;
Yuzhi11_06305	1	AP2+Alpha-Amylase Inhibitors (AAI), Lipid Transfer (LT) and Seed Storage (SS) Protein	Insect resistance; Abiotic stress response	Kumar et al., 2018a; Karray et al., 2022; Ming et al., 2022
Swetha_11540	4	40S ribosomal protein S15a, DNA translocase FtsK, AP2	Growth regulator; Chromosome segregation, Cell division; Oxidative stress	Mishra et al., 2022
Swetha_02245	5	Translation initiation factor 2B subunit, eIF-2B alpha/beta/delta family+ AP2	Plant virus resistance	Shopan et al., 2017
Swetha_02461	11	AP2+plasma-membrane proton-efflux P-type ATPase	Stomatal opening; Stress response	Ren et al., 2021; Mishra et al., 2022
Mishuozhima_25500	9	AP2+mito chondrial carrier prot (3 no.)	Stress recovery; Osmotic stress response	Monné et al., 2019
Swetha_28474	36	AP2+ Importin repeats (4 sets)+ HEAT like repeat+ Karyopherin (importin) beta	Autoimmunity; Pathogen response; Abiotic stress	Xu et al., 2020; Lüdke et al., 2021
Baizhima_11916	5	AP2+ zinc-binding in reverse transcriptase	Adaptive evolution; Stress response	Galván-Gordillo et al., 2016; Lanciano and Mirouze, 2018
Swetha_41017	8	AP2+AP2+Solute carrier families 5 and 6-like	Amino acid transport; Stress response	Hrmova and Gilliham, 2018
Yuzhi11_07044	16	alpha/beta hydrolases;+AP2+Ap2	Pathogenecity; Plant immune responses; Defense	Mindrebo et al., 2016; Jiao and Peng, 2018
Swetha_16136	9	alpha/beta hydrolases;+AP2+Ap2	Pathogenecity; Plant immune responses; Defense	Mindrebo et al., 2016; Jiao and Peng, 2018
Swetha_07942	11	AP2(4 dom)		
Swetha_02835	20	Protein FAR-RED ELONGATED HYPOCOTYL 3 (2 dom)+ FAR1 DNA-binding domain (Zn binding-2 dom)+ AP2	Negative regulation of carbon starvation and leaf senescence	Ma and Li, 2021; Tian et al., 2021
Swetha_18222	10	AP2+PWWP domain	Chromatin methylation reader; Stress response	Kenzior and Folk, 2015; Rona et al., 2016
Swetha_28399	14	Helicase+AP2	Plant stress response	Raikwar et al., 2015; Pandey et al., 2020
Swetha_28866	6	AP2+Tim17	Germination and Stress response	Chaudhuri et al., 2020
Swetha_03474	4	2 WRKY		
Swetha_14675	10	suppressor of G2 allele of SKP1+WRKY	Defense signalling and plant immunity	Yu et al., 2020
Swetha_21913	2	WRKY+MALA s1 propellar blade	Pathogenecity, Symbiosis	
Swetha_23996**^#^**	9	WRKY+Lung-7-transmembrane receptor	Plant immunity, Salt stress, Pathogenesis	Wu and Urano, 2018; Lu et al., 2019.
Swetha_18596	10	(WRKY+plant Zn cluster) 2 doms+ Signal recognition particle 9 kDa protein (SRP9)	Protection of mRNA degradation, Pathogen response	Bedassa et al., 2019; Kellogg et al., 2021
Swetha_00744	3	Protein kinases+C-terminal regulatory domain of Calcineurin B-Like (CBL)-interacting protein kinases+WRKY	Abiotic stresses like salt, drought, alkali	Pandey et al., 2015; Luo et al., 2017
Swetha_26866	6	Ubiquitin-protein ligase+WRKY	Abiotic stress response; Autophagy during stress response and development	Shu and Yang, 2017; Xu and Xue, 2019; Su et al., 2020
Swetha_26113**^(Y)^**	3	Arginine/lysine/ornithine decarboxylase+ WRKY	Abiotic stress response	Upadhyaya et al., 2021;
Swetha_39148	7	activating enzymes (E1) of the ubiquitin-like proteins+WRKY	Plant immunity, Autophagy during stress response	Su et al., 2020
Swetha_25903^#^	5	WRKY+DUF 3084		
Swetha_38328	10	Tellurite-resistance/Dicarboxylate Transporter (TDT) family_ SLAC1+ pepsin retropepesin -like aspartate proteases+WRKY	Regulation of stomatal movement; Stress adaptation; Plant immunity	Zhang et al., 2018; Deng et al., 2019; Sun et al., 2019
Swetha_36198**^#^**	11	ATP-dependent metalloprotease FtsH+ FtsH Extracellular+WRKY	Photosystem II repair; Photo-oxidative stress	Nixon et al., 2010; Kato and Sakamoto, 2018; Pu et al., 2022
Swetha_37469	9	RWP-RK domain+ Prot. Kinase+WRKY	Nitrate signalling pathways; Nitrogen stress; Nodulation	Ge et al., 2018; Mu and Luo, 2019
Swetha_09150	6	eukaryotic translation initiation factor 5A+WRKY	Pathogen response	
Swetha_09155	9	eukaryotic translation initiation factor 5A+WRKY+WRKY		
Swetha_13727**^(Z)^**	3	eukaryotic translation initiation factor 5A+WRKY+WRKY		
Swetha_24868	17	WRKY+WRKY+Chorismate mutase typeII+ effector domain of the CAP family of transcription factors+Voltage-dependent potassium channel	Salinity and drought, Negative regulation of salicylic acid pathway; Transcription activation; Electrical signalling; Stress response	Busby and Ebright, 1999; Wang et al., 2018; Poveda, 2020; Musavizadeh et al., 2021; Dreyer et al., 2021
Swetha_37939	9	WRKY+WRKY+WRKY+WRKY		
Swetha_19579	7	WRKY+Cyclopropane fatty-acyl-phospholipid synthase and related methyltransferases	Lipid transport and metabolism; Protection from herbivory	Yu et al., 2011; Okada et al., 2020
Swetha_04251	1	WRKY+peptide synthase	Oxidative stress response; Pathogen response; Plant-pathogen interaction	Golomb et al., 2018
Swetha_09533	14	galactokinase+plant heme-dependent peroxidase (Class-III)+WRKY	Carbohydrate metabolism, Abiotic stress signalling	Xiao et al., 2015; Stein and Granot, 2018

The fused domains, the number of introns in the gene, reported functions and references are given. The symbols **^#,Y^** and **^Z^** indicate present in all, present in all except Yuzhi11 and present in all except Zhongzhi13 respectively.

### 3.6 Alternative splicing of AP2/ERF and WRKY genes

**Figure 5:**
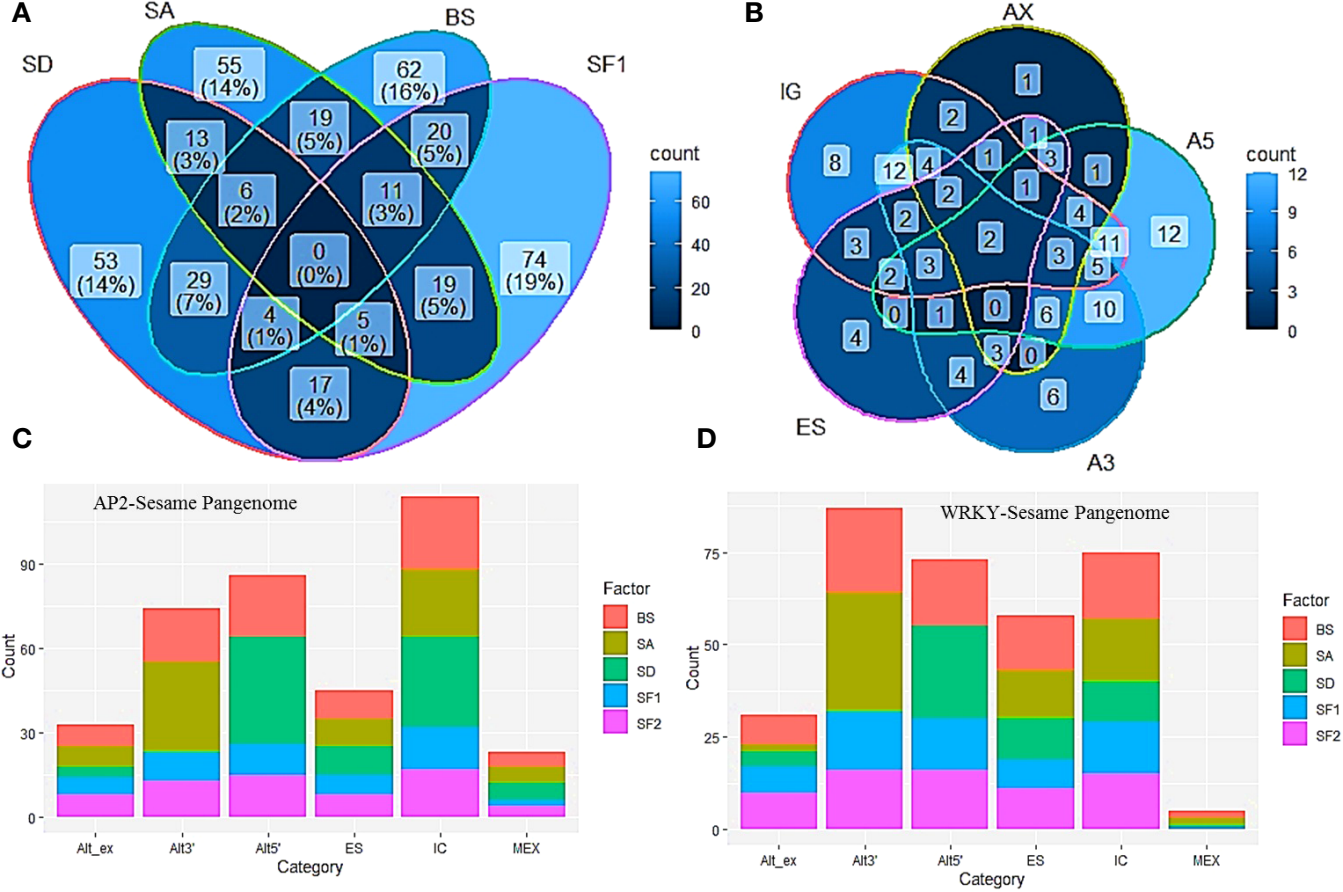
Alternative splicing in AP2/ERF and WRKY genes. **(A)** Venn diagram indicating major changes in Splice Acceptor (SA), Splice Donor (SD), Branch Site (BS) and the exonic splicing enhancer Serine/Arginine Splicing Factor 1 (SF1). **(B)** Venn diagram showing major splice mechanisms including Intron Gain (IG), Exon Skipping (ES), Alternative 5’ ends (A5), Alternative 3’ ends (A3) and Alternative exon ends (AX). **(C, D)** indicate changes observed in splice enhancers and branch sites in AP2/ERF and WRKY respectively.

The occurrence of multi-domain genes, protein variants and variable exon-intron structures of homologs in the sesame pan-genome prompted a thorough study of splice junctions to identify the splice variants in the pan-genome. After comparing exon-intron structure and the splice junction scores between the most common homolog and the identified variables we categorized the splice variants into different alternative splicing events. (**SI-5, SI-7**). We found the occurrence of the following splice events in the pan-genome namely intron gain or loss, alternative exon ends, alternative 5’ and 3’ ends, mutually exclusive exons, exon skipping and intron retention. We found splice variants in 70 and 52 genes of AP2/ERF and WRKY families respectively (**Figure 5A**). Multiple AS events were noticed in many cases and in AP2/ERF gene family intron gain or loss was most common followed by alternative 5’ and alternative 3’ events. The least common was intron retention and mutually exclusive introns. In the WRKY family, alternative 3’ followed by alternative 5’ ends were the most preferred splice variant. Here also intron retention and mutually exclusive introns were less common. (**Figure 5B**).

With respect to each of the variants, a corresponding change in both position and score was noticed in the splice acceptor site, splice donor site, branch site as well as different splicing enhancers. Although characterized majorly in human alternative splicing scenario, SF2 and SF1 are reported to have major roles in plants especially during salinity and irradiation stress (Stankovic et al., 2016; Jin, 2022). Among the AP2/ERF genes of sesame, splice donor site mutations were majorly reflected in alternate 5’ ends, intron gain and exon skipping events in the genome. Spice acceptor site mutations drastically altered 3’ ends added by intron gain and exon skipping. Branch site mutations complemented most of the AS events although majorly reflected in intron gain. Mutation in all of the splice enhancer element positions and sequences supported different AS events. Changes in SF1 majorly affected intron gain or loss whereas SF2 mutations helped in modifying 5’ and 3’ ends of exons in addition to intron gain. The mutations in SF2 favored alternate 5’ ends. Alternate exons and mutually exclusive exons involved mutations in splice donor, acceptor and the branch sites (**Figure 5C**). In the WRKY family, the change in splice donor site was reflected in alternate 5’end, intron gain and exon skipping. The splice acceptor site change was noticeable in alternate 3’ ends, intron gain and exon skipping. The branch site changes were observed in all the splice events recorded. Intron gain was associated with SF1and SF2 whereas alternative exon ends, exon skipping and alternative 5’ ends were majorly associated with SF2. Mutually exclusive exons were comparatively less in the WRKY family and were associated with changes in splice acceptor, branch site and SF1 (**Figure 5D**).

### 3.7 Synteny and collinearity in sesame pan-genome

The comparison of the ‘Swetha’ and arabidopsis genomes resulted in the identification of 981 syntenic blocks involving 18614 genes with a collinearity percentage of 26.78 (**Figure 4B**). With rice, the collinearity percentage was 7.08 and 5947 collinear genes were detected in 451 blocks (**Figure 4C**). The rice chromosomes 11 and 12 did not contain any syntenic homologs of sesame WRKY and AP2/ERF genes. Moreover, 103 whole genome or segmentally duplicated AP2/ERF and WRKY genes are retained as syntenic blocks in Arabidopsis compared to 48 of rice. The comparison of genomes of Zhongzhi-13 and Swetha revealed extensive synteny and collinearity among sesame genes. Of the total 78048 genes present in the two genomes, 48729 were found to be collinear. The percentage of collinearity was 62.43 and 956 syntenic blocks were present (**Figure 4A**). WGD/segmental duplication was found in 175 genes including 73 WRKY and 103 AP2/ERF genes in Swetha. The segmental duplication genes in Zhongzhi-13 included 108 AP2/ERF and 69 WRKY genes. Synteny analysis revealed the presence of three single-copy WRKY genes in the Chinese accessions including the homologs of Zhongzhi13_22905, Zhongzhi13_26827 and Zhongzhi13_29190. Details are in **SI-9**.

To study the selection pressure during evolution, Ka/Ks statistics were worked out. Significant selection effects were noticed on 70 pairs of AP2/ERF genes and 54 pairs of WRKY genes in the sesame pan-genome. In the AP2/ERF family 26 gene pairs were under purifying selection (Ka/Ks <1), 45 were under positive selection (Ka/Ks>1) and 2 pairs were under neutral selection (**Figure 4D**). Among the 54 duplicate genes under selection in the WRKY gene family, 29 were under purifying selection while positive selection effects were noticed in 25 pairs (**Figure 4E**). We also compared the selection effects of duplicated genes in Swetha and Zhongzhi-13, as well as the gene copy number varaiants of Swetha with each other (**Figure 4F**). Between the two genomes 52 AP2/ERF and 71 WRKY genes were under selection pressure. In the AP2/ERF family, 26 gene pairs were under positive selection while 26 were under purifying selection. The number of segmental duplicates under positive selection was much higher than dispersed genes whereas an equal distribution was found for purifying selection. Among the WRKY genes, 28 gene pairs were under purifying selection while 43 were under positive selection. The segmental duplicated genes were under severe selection pressure in both categories. Among the 41 duplicated gene pairs identified in Swetha, selection effects were significant for 22 pairs. Here also the number of gene pairs under positive selection was higher than that under purifying selection SI-10 and SI-11. The evolutionary time period in million years for AP2/ERF genes was 0.4-143 and for WRKY genes was 0.8-137. The genes under neutral selection in AP2/ERF family were most recently evolved.

### 3.8 Protein-protein interactions, functional domains and subcellular localization

**Figure 6:**
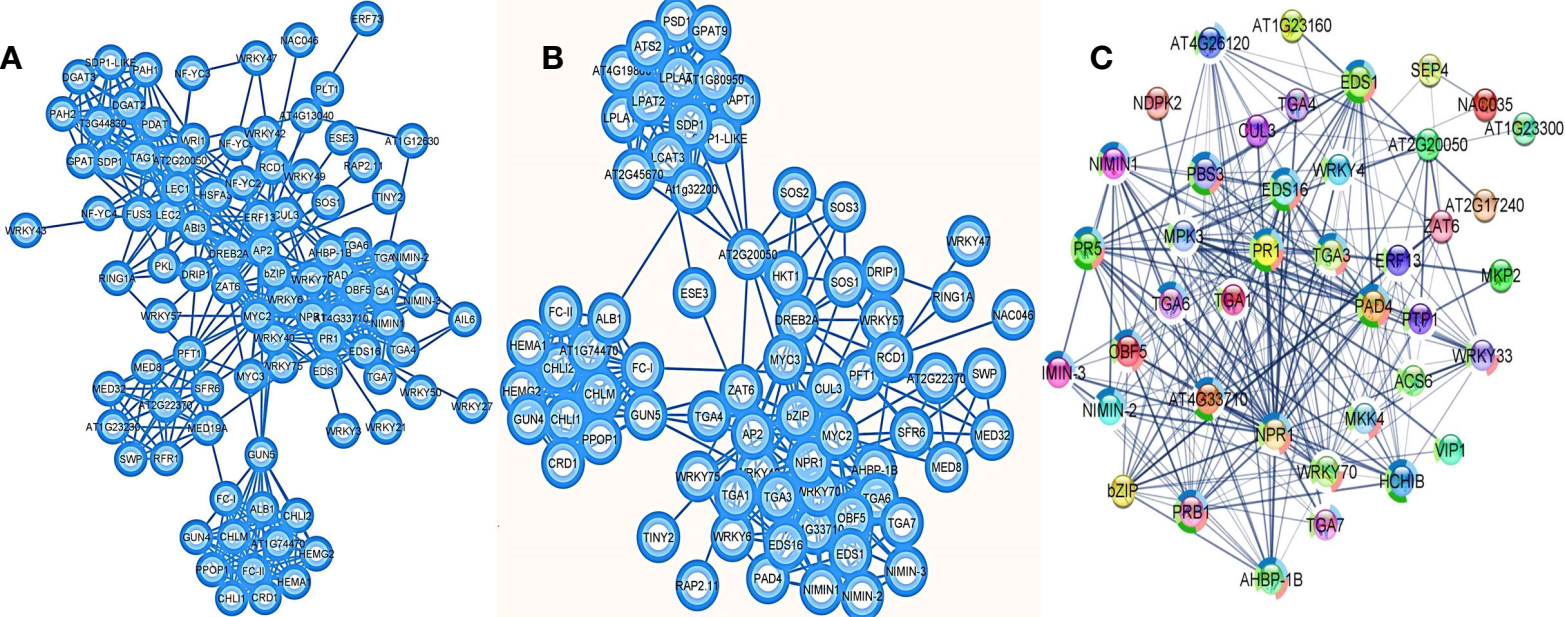
Protein-protein interactions of AP2/ERF and WRKY genes in **(A)** Arabidopsis homologs and **(B)** Sesame genome. In sesame defense response is closely related with lipid metabolism and chlorophyll biosynthesis. **(C)** Represent the interactions of WRKY fusion gene, Swetha_24868 as a master regulator of defense signaling in the absence of NBS-LRR genes in sesame.

The protein-protein interaction network visualized the major roles of AP2/ERF and WRKY transcription factors in defense mechanism, stress response, lipid metabolism and chlorophyll biosynthesis (**Figures 6A, B**; **SI-16**). The major interaction partners for defense and stress response included NIMIN family genes, TGA transcription factors, WRKY genes, MEDIATOR family genes including AT2G22370, PAD4 (Phytoalexin deficient 4), EDS1 (Enhanced Disease Susceptibility1), HKT1 (High-Affinity K^+^ Transporter 1), bZIP family, MYC, ZAT family, Putative E3 ubiquitin-protein ligase RING1a, Cullin homolog 3 (CUL-3), ethylene activated signaling pathway genes like DREB, TINY, RAP, etc. In chlorophyll biosynthesis, the major interaction partners were CRD1 (Copper Response Defect1), GUN (Genomes Uncoupled) 4 and 5, FC (Ferrochelatase) I and II, Albina1, Mg-protoporphyrin chelatase different sub-units (CHLI1, CHLI2, CHLM), Geranylgeranyl reductase (GGR, given as AT1G74470), Glutamyl-tRNA reductase 1 (HEMA1) and Protoporphyrinogen oxidase 1 (PPOP1). Among the lipid metabolism pathway genes, the major interactions identified were with lecithin–cholesterol acyltransferase (LCAT), lysophosphatidic acid acyltransferase (LPAT2), triacylglycerol lipase, Sugar-Dependent1 (SDP1), lysophosphatidylcholine acyltransferase (LPCAT), lyophosphatidylethanolamine acyltransferase (LPEAT1/AT1G80950 and LPEAT2/AT2G45670), Glycerol-3-phosphate acyltransferase 9 (GPAT9), phosphatidylserine decarboxylase (PSD1), Glycerol-3-phosphate acyltransferase (ATS1/AT1G32200), etc. All three pathways showed significant interaction with AT2G20050 representing cAMP-dependent protein kinase involved in the PKA signaling pathway. Swetha_24868, a gene functionally similar to WRKY 4 and Zinc dependent activator protein1 of Arabidopsis was found to act along with WRKY 70 and WRKY 33 initiating cascades of different defense responses (**Figure 6C**). Many interaction partners were observed to have significant roles in systemic acquired resistance (SAR), osmotic stress, hypoxia, cold stress and pathogenesis.

The significant GO terms associated with AP2/ERF and WRKY transcription factors include cellular response to stress, defense, immune response, intracellular signal transduction, MAPK cascade, regulation of the cellular process, response to abiotic stress, systemic acquired resistance, salicylic acid-mediated signaling pathway, plant-pathogen interaction, ribosome assembly, ribosome biogenesis, translation, etc. The conserved motifs identified through MEME (SI-13A) for WRKY family had molecular functions like transcription factor activity, ATPase activity coupled to transmembrane movement of substances and structural constituent of ribosome while associated with cellular components of the mitochondrion, ribosome and chloroplast (stroma, thylakoid and envelope). The biological process identified was translation. For the AP2/ERF family (SI-12A) the molecular functions attributed were transcription factor activities, structural constituent of ribosome, protein serine/threonine kinase activity and protein binding while being part of biological processes like translation, protein amino acid phosphorylation, transmembrane receptor protein tyrosine kinase signaling pathway and glycolysis. The cellular components identified were the nucleus, mitochondrion, chloroplast, ribosome and cullin-RING E3 ligases (CRLs) complex.

The subcellular localization was studied in detail to understand the regulatory functions. 56% of WRKY and 60% of AP2/ERF transcription factors had a high probability of being located in the nucleus. Among the WRKY genes 20% were predominantly localized in the chloroplast, 7.9% in the cytoplasm, and 6.4% in the mitochondria. For AP2/ERF family the statistics were 17%, 3.2% and 10.89% respectively (SI-12B). The WRKY gene Swetha_04277 and its three homologs were localized in the peroxisome whereas the Baizhima gene (Baizhima_17686) was localized in the chloroplast (SI-13B). Similarly, Zhongzhi13_00117 and three Chinese homologs were located in the extra-cellular space whereas Swetha_00675 was located in the nucleus. Among the splice variants, 64% of WRKY and 70% of AP2/ERF homologs showed differential sub-cellular localization.

### 3.9 Cis-element analysis in the promoter regions of AP2/ERF and WRKY genes

We examined the cis-element sequence distribution in selected single exon genes, all the splice and exonic variants of AP2/ERF genes and selected homologs from all the WRKY classes from Swetha and Zhongzhi-13. In addition to the common cis-acting elements CAAT box and TATA box, elements regulating phytohormonal response, development and stress response were found. More than 75% of identified cis-elements responded to abiotic and biotic stress responses including drought, salinity, light and pathogenesis. The most common cis-elements identified were ABRE, as-1, MBS, MYB, MYC and STRE. MYB and STRE were present in all the WRKY genes while MYC was present in all the AP2/ERF genes used for the study. Very few instances were found where the conservation existed all through the exon, intron and regulatory regions among the homologs as evident in Swetha_30858 & Zhongzhi13_23474 and Swetha_24927 & Zhongzhi13_18440 from AP2 family. Among gene duplicates in the same genome with difference in exon-intron size or numbers, one gene is found to retain similarity to parental regulatory sequences. Examples include Swetha_18219 & Swetha_18222, Swetha_28864 & Swetha_28866, Swetha_33069 & Swetha_33068, Zhongzhi13_33595 & Zhongzhi13_33551 and Swetha_6527 & Swetha_3532 (WRKY). Even in genes with a single intron and conserved protein structures, the regulatory landscape varied widely. In many cases, in spite of similar protein structures, a drastic change was noticed in the number of a core promoter like TATA to the extent of 2-3 folds, like in Swetha_04095 & Zhongzhi13_02825 and Swetha_41134 & Zhongzhi13_32245 indicating dosage effect. Details are in **Figure 3A–C**, **SI-14, SI-15**.

### 3.10 In-silico gene expression profiles of AP2/ERF and WRKY gene families

**Figure 7:**
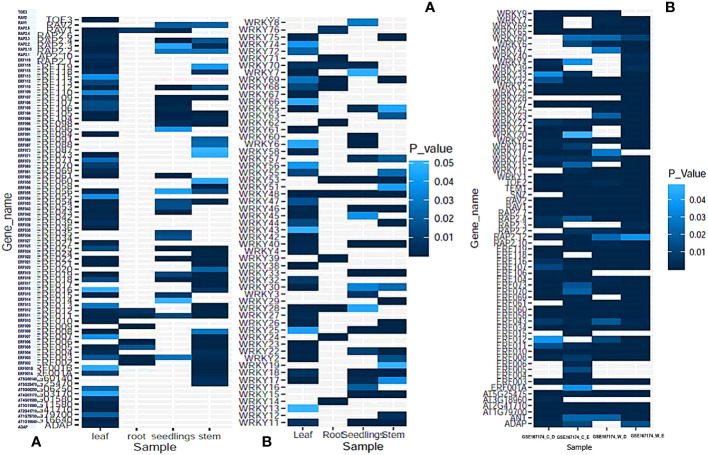
The expression profiles of AP2/ERF and WRKY genes during **(A)** Abiotic stress response in different tissues and seedlings. **(B)** During wounding response and biotic stress response in WIND1 mutants and wild type plants. P-values were calculated based on Welch’s t-test and genes with P-value ≤0.05 is represented either up regulated or down regulated (> 2-fold or < 0.5-fold).

The GEO profiles targeting different biotic and abiotic stresses like drought, heat, salinity, osmotic stress, cold stress, wounding, etc. and primary cell wall thickening was selected with expression sites at stem, roots, leaves and seedlings. AP2/ERF genes ERF003 and ERF011 as well as WRKY48 were found to express under all types of abiotic stress and in all the tissues.WRKY48 was found up-regulated in the stem, seedlings and root while down-regulated in roots. 8 AP2/ERF and 12 WRKY genes were found down-regulated in the roots including ERF003, ERF005, WRKY76, WRKY62 and WRKY24. Most of the AP2/ERF family genes were found to express in the leaves and stem (SI-17A). 6 AP2/ERF genes involved in primary cell wall thickening including ERF034 and ERF043 and were found to express in leaves and seedlings. 10 AP2/ERF and 5 WRKY genes including WRKY-4, 7 and 74 were found down-regulated in leaves during abiotic stress. As for biotic stress response, 22 differentially expressing AP2/ERF and WRKY genes were detected in response to wounding. Up-regulation was noticed in RAV2, ERF070, ERF060, RAP2.4, ERF011, ERF107, WRKY29, TEM1, ERF034, WRKY32, WRKY27, ERF118, RAP2.7, WRKY22, WRKY16, WRKY7 and ERF012 with more than 2 fold changes. 10 genes were down-regulated including WRKY39, WRKY69, ERF104 and ERF116. The expression levels of AP2/ERF and WRKY genes are given in **Figure 7A, B**, **SI-17 B-D**.

## 4 Discussion

Sesame grown in marginal environments across the world is subjected to various kinds of abiotic stresses like drought, water logging and salinity. Recently a pan-genome was developed by combining genomic sequences of five cultivars (Yu et al., 2019) including the reference genome Zhongzhi-13 (space mutant), Chinese landraces Baizhima and Mishouzhima, major Chinese domestic cultivar (Yuzhi11) and the Indian variety ‘Swetha’. Pan-genome offers a better understanding of the evolutionary mechanisms that allow organisms to adapt faster to changing environments (Tranchant-Dubreuil et al., 2019).

Environmental adaptations change the genomic architecture and result in the introduction of new genetic diversity into elite cultivars which can be accessed through genome sequencing. Plant adaptations mainly rely on Structural Variations (SVs) including Presence/Absence Variations (PAVs) and Copy Number Variation (CNV), particularly for biotic and abiotic stress tolerance (Khan et al., 2020). Our focus was on the evolutionary adaptations pertaining to maximum fitness among the component genomes adapted to a wide ecological niche. In the major regulators of signal transduction and gene expression under biotic & abiotic stress conditions, AP2/ERF and WRKY, variation was detected in gene number, exon and intron numbers and size, protein characteristics, location in the genome, and promoter sequence architecture.

The number of AP2/ERF genes detected in wheat (322), sorghum (122), rice (139), Arabidopsis (122), *Brassica napus* (531) and sugarcane were comparable to sesame as per the ploidy level (Ghorbani et al., 2020; Riaz et al., 2021; Li et al., 2021). The numbers of RAV and soloist family members were much higher than those reported in soybean, rice and Arabidopsis while comparable with that of pear (Li et al., 2018). The number of AP2/ERF genes reported in the pan-genome (145) is higher than earlier reports (132) by Dossa et al., 2016 in sesame with wide difference in classification. In the WRKY family, we detected 89 genes in the pan-genome, much lesser than reported in sorghum (94), rice (104), Arabidopsis (74), apple (113), soybean (174) and sunflower (119) (Yang et al., 2017; Baillo et al., 2020; Liu et al., 2020; Abdullah-Zawawi et al., 2021; Qin et al., 2022). Like other crops, in the sesame pan-genome class-II WRKY’s dominated the family whereas the class-III members were very less, 5 in individual genome against 15 and 31 in rice and sorghum respectively. The earlier reports on sesame (Li et al., 2017) suggested 71 WRKY genes in sesame with 7 in class III.

Dossa et al., 2016 reported that 70% of AP2/ERF genes are intron-less and the exons detected were 1-10 in the sesame genome. In the pan-genome we found 63% of AP2/ERF genes to be intron-less with the numbers changing drastically with the individual genome. 53.1% of genes of Swetha genome were intron-less whereas the landraces, Baizhima and Mishouzhima had higher amounts, 67 and 68% respectively. More than 10 exons were detected in 16 genes covering all the genomes while the bulk was contributed by Swetha (7 genes). The number of exons also varied drastically across the genome with the Swetha genome having 3.5 exons/gene as against 2.6 in the pan-genome. In the sorghum pan-genome, 4.2 exons/genes were reported (Tao et al., 2021). Similarly in the WRKY family, the Swetha genome contributed 5 exons/gene as against 4.2 in the pan-genome. Li et al., 2017 reported sesame WRKY genes to consist of 1-11 introns whereas two genes with 14 and 17 introns were detected in the Swetha genome. Similarly, the introns/gene for WRKY and AP2 genes are 3.21 and 1.7 respectively whereas for Swetha it was 4.0 and 2.45 respectively, as against 4.15 introns/gene in plants (Frey and Pucker, 2020). The AP2 sub-family genes had 2-20 introns with the majority of genes having 5-8 introns. Dossa et al., 2016, reported the intron numbers to be 3-10 for AP2 and a single intronic gene was also identified. The ERF sub-family was found to be intron poor with 95% of members having 0-1 introns in accordance with earlier reports. The gene, Swetha_28474 had 36 introns as against the maximum of 9 introns reported earlier (Su et al., 2022). In the WRKY family, the intron numbers reported vary between 0-5 in rice (Abdullah-Zawawi et al., 2021), 0-11 in eggplant (Yang et al., 2020), 1-6 in barley (Zheng et al., 2021) and 1-11 in sesame (Li et al., 2017). One 11 intron T2C sub-family gene was found to be conserved in the genome without any change in exonic sequences although size variation was noticed for the 2^nd^ intron. Enhanced gene copy number, as well as the predominance of large genes with multiple exons in the genome of Swetha, was reported earlier for TCP (Parakkunnel et al., 2020) and HSF (Parakkunnel et al., 2022) gene families in sesame.

A similar trend was found in the case of micro-exons wherein family-wise difference was quite wider with AP2/ERF family having more micro-exons than WRKY. The AP2/ERF family was reported to be micro-exon rich (Song et al., 2020) and in the sesame pan-genome, micro-exons totaled 10% of total exons as against 1.96% in the WRKY family. 88% of micro-exons were contributed by the AP2 subfamily in the sesame pan-genome with only 15 out of 128 identified genes lacking micro-exons. Song et al., 2020, also reported that AP2 micro-exon genes are highly conserved which we found only to be partially true in the pan-genome. 44% of micro-exon genes were conserved in the pan-genome in the AP2 sub-family whereas the extent was higher in the Chinese accessions particularly in landraces Baizhima and Mishouzhima, wherein 83% sequence conservation was observed. WRINKLED1 (*WRI1*) is an AP2 gene widely studied in Arabidopsis and higher plants (Ma et al., 2013) acting as a master regulator of fatty acid synthesis. The presence of 9bp long micro-exon coding for amino acids ‘VYL’ and its isoforms have been reported as essential for the *AtWRI1* gene. This micro-exon was missing in the sesame pan-genome. Instead, the WRI1 homologs of sesame may be alternative splice forms of the gene as reported in castor (Ji et al., 2018). The sesame homologs lacked ‘VYL’ sequence and formed five different clusters with intron numbers ranging from 4-20 although Yuzhi11 and Zhongzhi13 genomes shared the sequence (SI-13). The atypical splicing combined with the skipping of conserved micro-exons resulted in unique *WRI1* genes in rice (Mano et al., 2019). In the pan-genome (SI-4) exon skipping, gene fusion, mutually exclusive exon, as well as alternative 5’ and 3’ splice ends contribute to the generation of novel variants in *WRI1* genes. Micro-exons of size ≤15bp are considered as shortest and interestingly 38% of WRKY micro-exons belonged to this category whereas in the AP2/ERF family only 9% of micro-exons fulfilled this criterion (Yu et al., 2022). The most common micro-exons occurring in AP2/ERF genes are of sizes 9, 26, 31, and 45 bp according to Song et al., 2020. In sesame, the most common micro-exon is 50 bp occurring in 54% of AP2 genes at exonic positions 4, 5 or 6. An estimated 35% of AP2 genes without the 50bp micro-exon contained two micro exons of 25 and 30 bp size, occurring together at 2^nd^ and 3^rd^ exons in the same order. A 44bp micro-exon also occurred in the same group together with 25 and 30 bp micro-exons in 50% of cases. Only one case of exclusion of 25bp micro-exon was noticed in the sesame pan-genome wherein gene fusion involving PWWP domain with AP2 domain was observed in Swetha_18222 drastically altering genic architecture.

Among the 26 WRKY genes with micro-exons in the sesame pan-genome, 15 genes (all belonging to Chinese accessions) did not show any variation in exon-intron structure. However, alternative splice forms were noticed in 11 WRKY genes with micro-exons with more than 80% belonging to the Swetha genome. In the AP2/ERF family, alternative splice forms were noticed in 47 micro-exon genes (35%); while the majority (47%) again belonged to Swetha. Altogether, in the Swetha genome, 49% of genes with micro-exons were alternative splice forms. It can be understood (**Table 2**) that micro-exons are important functionaries of stress tolerance, circadian clocks, apomixis, defense response, the integrity of genetic material and transposon integration (Liu et al., 2013; Theriault and Nkongolo, 2017; Aguado and tenOever, 2018; Curtis et al., 2018; Wu and Urano, 2018; Worthington et al., 2019; Wang et al., 2019; Shi et al., 2020; Song et al., 2020; Tischkau, 2020; Chen et al., 2021; Ming et al., 2022.)

However, we do not agree with Song et al., 2020 that the micro-exons of AP2 genes are under negative selection. In the pan-genome, 4 genes were under neutral selection; 11 were under purifying selection, and 25 were under positive selection. Genes under positive selection included micro-exons with domain similarities to lecithin: cholesterol acyltransferase (LCAT3), aquaporin and integrase while the ASGR-BBM-like micro-exon gene was under neutral selection. Most of the micro-exon genes (>65%) were also under WGD or segmental duplication.

Gene fusion was identified as a potent source of creation of evolutionary novelty wherein a new gene arises by joining parts from two or more genes and is controlled by the same regulatory region. It is widely reported in bacteria (Pasek et al., 2006) as the evolutionary force behind multi-domain proteins and in eukaryotes resulting in cancer-genesis mutations with deleterious consequences (Glenfield and Innan, 2021). In higher plant genomes gene fusion is a complex process with a poorly understood evolutionary mechanism. Gene fusion events have been reported in the biosynthesis of alkaloids in opium poppy (Catania et al., 2022), viral infection in maize (Zhou et al., 2022a) and in the evolution of new genes in the genus Oryza (Zhou et al., 2022b). In the sesame pan-genome, 2.3% of AP2/ERF genes and 10% of WRKY genes were products of gene fusion. Larger share of these novel variations was contributed by the Swetha genome where 8.3% of AP2/ERF and 24% of WRKY genes arose due to gene fusion. In the Oryza genus, the *O*. *japonica* genome had more fusion genes compared to *O. indica*, *O. barthii* and *O. glaberrima* (Zhou et al., 2022b) whereas differential gene fusions were observed among different Papaver species affecting alkaloid concentration (Catania et al., 2022). However, the gene fusions reported in sesame, particularly in Swetha are much higher than in maize (Zhou et al., 2022a) and Oryza (Zhou et al., 2022b). The duplication of parental genes prior to fusion was evident also in the sesame genome (Zhou et al., 2022b) while most of the added domains were products of horizontal transfer. In sesame, gene fusion and creation of novel multi-domain genes were reported in HSF genes (Parakkunnel et al., 2022) while the fusion with retrotransposons were reported for TCP genes (Parakkunnel et al., 2020). Selection effects were significant for 21 fusion genes in sesame wherein positive selection was noticed in >71% of genes in contrast to the rice genome (Zhou et al., 2022b).

Alternative Splicing (AS) increases the complexity of the transcriptome and proteome by generating multiple transcripts of the same gene through differential processing of introns and exons in pre-mRNA (Martín et al., 2021). Reports suggest that AS events are involved in the regulation of gene expression under a multitude of environmental and biotic stresses (Laloum et al., 2018: Martín et al., 2021). The identified spice variants in AP2/ERF and WRKY genes were 70 (48.27%) and 52 (58.43%) respectively with maximum splice variants per single gene of 6 and 7. Swetha genome contributed 40-50% of these variants.

Majority of the exonic additional domains and micro-exonic domains were involved in abiotic stress responses as well as hypersensitive responses against plant pathogens (**Tables 2**, **3**). The maintenance of cell membrane and organelle integrity under stress is majorly dependent on the lipid and carbohydrate composition of the cell dynamically affecting the performance of cellular transporters (Rawat et al., 2021). Moreover, the epigenetic regulation of stress response through chromatin remodeling, regulatory RNA’s and DNA methylation is manifested in the additionally acquired domains (Chinnusamy et al., 2013). Reactive Oxygen Species (ROS) are produced at higher levels in different organelles during abiotic stress and cause significant damage to the cell. Ironically ROS is also essential for stress response as they act as signaling molecules triggering signal transduction (Frederickson Matika and Loake, 2014). Plant FtsH4, an ATP-dependent mitochondrial protease is essential to preserve stem cell activity and temperature stress response throughout development and lack of which leads to the precocious cessation of growth (Huang et al., 2019). An important component of ROS signaling, FtsH4 is associated with photosystemII repair mechanism and mitigation of photo-oxidative stress (Khanna-Chopra, 2012) and manifested through apoptosis and leaf senescence. It is important that a fusion gene with WRKY and FtsH is conserved across the pan-genome indicating adaptive evolution under stress conditions. Similarly, E3 ubiquitin ligase essential for less accumulation of ROS and enhanced anti-oxidant capacity under drought stress, existed in fusion with the WRKY gene in sesame (Zhang et al., 2017). Cullin-RING E3 ligases (CRLs) were identified as a major cellular component in meme analysis. Oxidative burst; wherein ROS accumulation leads to the production of hydrogen peroxide (H_2_O_2_) occurs as a defense response against pathogens. Aquaporins help the diffusion of H_2_O_2_ through the plasma membrane to cytosol triggering MAMP-triggered immunity (Bigeard et al., 2015).

WRKY and AP2/ERF genes are expressed at all parts of the plant; root, stem, leaves and seedlings and in addition to stress response also function in the light-induced stomatal opening, redox homeostasis, callus formation, starch metabolism, cell wall biosynthesis, RNA regulation of transcription, hormone metabolism and lignin biosynthesis. The multi-domain fusion genes are involved in all the major defense pathways like SA-mediated signaling, and MAP kinase cascades (Eulgem and Somssich, 2007). WRKY genes are known to activate sugar-responsive genes through an epigenetic mechanism and a fusion gene Swetha_09533 containing the galactokinase domain is directly involved in sugar metabolism (Chen et al., 2019). Particularly the additional domains of the T1 WRKY gene Swetha_24868, chorismate mutase, cAMP and voltage-dependent potassium channel convert this gene into a master regulator. Recruiting other WRKY and AP2/ERF genes into the picture, Swetha_24868 mediates defense responses involving Salicylic acid-dependent signaling, thiol-based signaling and MAPK signaling. Chorismate mutase regulates defense mechanisms through enhancing the accumulation of SA, lignin and antioxidants (Jan et al., 2021). The cAMP (3′, 5′-cyclic adenosine monophosphate) is known as an important signaling molecule in defense responses in addition to roles in germination, stomatal opening, ion homeostasis and cell cycle progression (Blanco et al., 2020). Voltage-gated K+ channels are involved in high salinity stress and maintaining ion homeostasis in sweet potato (Zhu et al., 2022) and rice (Musavizadeh et al., 2021). Interestingly in the absence of NBS-LRR genes in sesame, the pathway involves EDS-1, EDS-16, WRKY-4, NPR-1, PRB-1, PR-1, PR5, HCHIB and GH3 (AT1G23160) functioning in SA signaling pathway. In addition to triggering immunity, SA mediated pathway is also effective in regulating ROS levels as revealed by interaction with ERF-13 and CUL-3 (**Figure 6**). However, this gene and the parental gene, Swetha_24865 did not vary much in the promoter sequence except for the ABRE sequence in Swetha_24865. However, they did share DRE, MYB and MYC sequences. A single copy of DRE is needed for ABA independent induction of osmotic and cold stress genes and promoter sequence containing DRE without ABRE is found to work well under stress conditions (Yamaguchi-Shinozaki and Shinozaki, 1994). The localization of Swetha_24868 was found to be predominantly in chloroplast while Swetha_24865 was expressed equally in the nucleus and chloroplast. Class T3 of WRKY genes had W-box sequences in the promoter wherein protein localization, exonic and other cis-element sequences varied widely with individual genes. T2E genes, Swetha_21917 and Swetha_21913 as well as AP2 genes Swetha_33068 and Swetha_33069 in spite of sharing the same promoter sequences are located nearby each other and products of recent gene duplication localized differently in chloroplast and nucleus, respectively. The exon-intron, promoter sequence, localization and active domain diversity indicate the faster evolution scenario of defense response genes in sesame subjected to multiple stresses on account of its marginal growing conditions.

## 5 Conclusions

Breeding crop varieties for changing climate scenarios with the effective use of existing diversity is the primary challenge for food security. However, breeding techniques and adaptation to the environment significantly alter the genomic structure of crop plants. This was evident from the pan-genome study of sesame including varieties or landraces adapted for vastly varying climates of India and China. Although a certain level of gene conservation existed at the species level; evolution created different footprints on different genomes. The adaptive selection was evident in copy number variation and modification of function for most of the gene loci studied. Retention of ancient genes with the incorporation of extra functional domains to cope with extreme stress conditions was observed in *S. indicum.* Ecological adaptation was manifested in genome composition with geographical regions harboring variant forms of gene loci offering maximum fitness.

## Data availability statement

The datasets presented in this study can be found in on line repositories. The pan-genome data (Yu et al. 2019 ) is available in public domain and all the other data can be found in the supplementary materials. The Swetha genome was sequenced by a team involving KVB and RP and is deposited as bio-project ''PRJNA219369'' and assembly ASM97556v1. The other genomes are available at accession numbers GCA_000512975.1, GCA_003268515.1 and GCA_026168435.1.

## Author contributions

RP: Conceived the idea, extracted data, investigation, resources, softwares, analysed data, and drafted manuscript. BK: Investigation, review and drafted the manuscript. VG: Resources, review and writing. SC: Investigation, resources and drafted the manuscript. SP: Extraction of genic sequences. UK: Project administration, resources KB: Genomic resources, Swetha genome sequencing, manuscript finalization; SK: Supervision, project administration and manuscript finalization. All authors contributed to the article and approved the submitted version.

## Supplementary material

Data Sheet 1 Contains SI-18 (Fig). The AP2/ERF and WRKY genes mapped to the 13 chromosomes of sesame genome. Here the WRKY and AP2/ERF genes from Swetha genome have been represented in ‘blue’ and ‘red’ coloured fonts respectively. Please adjust the zoom to 100% for clear visibility.

Data Sheet 2 Contains SI-19 (Fig). The sub-families are coloured as per Swetha AP2/ERF classification given in Fig-2. The colour codes are as following: Blue=DREB; Magenta=ERF; Red=AP2; Green=RAV and Cyan=SOLOIST. Individual Swetha genes are coloured as per the initial classification of Fig-2 to reflect the extent of domain changes in the pan-genome during evolution.

Data Sheet 3 Contains SI-20 (Fig). Different groups are coloured as per classification given in Fig-2. T1=Blue; T2A= Orange; T2B=Magenta, T2C=Red; T2D=Lime yellow; T2E= Purple and T3= Olive.

## References

[B1] Abdullah-Zawawi M. R. Ahmad-Nizammuddin N. F. Govender N. Harun S. Mohd-Assaad N. Mohamed-Hussein Z. A. (2021). Comparative genome-wide analysis of WRKY, MADS-box and MYB transcription factor families in arabidopsis and rice. Sci. Rep. 11, 1–18. doi: 10.1038/s41598-021-99206-y 34608238 PMC8490385

[B2] Aguado L. C. tenOever B. R. (2018). RNase III nucleases and the evolution of antiviral systems. BioEssays 40 (2), 1700173. doi: 10.1002/bies.201700173 29266287

[B3] Baillo E. H. Hanif M. S. Guo Y. Zhang Z. Xu P. Algam S. A. (2020). Genome-wide identification of WRKY transcription factor family members in sorghum (*Sorghum bicolor* (L.) moench). PloS One 15 (8), e0236651. doi: 10.1371/journal.pone.0236651 32804948 PMC7430707

[B4] Bedassa S. B. Akkaya M. S. Ersoy F. (2019). HvSRP72 silencing enhanced *Blumeria graminis f.* sp. *hordei* growth in compatible interaction with barley. J. Plant Pathol. 101, 91–96. doi: 10.1007/s42161-018-0145-4

[B5] Bedigian D. (2003). Evolution of sesame revisited: domestication, diversity and prospects. Genet. Resour. Crop Evol. 50, 779–787. doi: 10.1023/A:1025029903549

[B6] Bigeard J. Colcombet J. Hirt H. (2015). Signaling mechanisms in pattern-triggered immunity (PTI). Mol. Plant 8, 521–539. doi: 10.1016/j.molp.2014.12.022 25744358

[B7] Blanco E. Fortunato S. Viggiano L. de Pinto M. C. (2020). Cyclic AMP: A polyhedral signalling molecule in plants. Int. J. Mol. Sci. 21, 4862. doi: 10.3390/ijms21144862 32660128 PMC7402341

[B8] Bouckaert R. Vaughan T. G. Barido-Sottani J. Duchêne S. Fourment M. Gavryushkina A. . (2019). BEAST 2.5: An advanced software platform for Bayesian evolutionary analysis. PloS Comput. Biol. 15, e1006650. doi: 10.1371/journal.pcbi.1006650 30958812 PMC6472827

[B9] Busby S. Ebright R. H. (1999). Transcription activation by catabolite activator protein (CAP). J. Mol. Biol. 293, 199–213. doi: 10.1006/jmbi.1999.3161 10550204

[B10] Catania T. Li Y. Winzer T. Harvey D. Meade F. Caridi A. . (2022). A functionally conserved STORR gene fusion in papaver species that diverged 16.8 million years ago. Nat. Commun. 13, 1–11. doi: 10.1038/s41467-022-30856-w 35672295 PMC9174169

[B11] Chaudhuri M. Darden C. Soto Gonzalez F. Singha U. K. Quinones L. Tripathi A. (2020). Tim17 updates: A comprehensive review of an ancient mitochondrial protein translocator. Biomolecules 10, 1643. doi: 10.3390/biom10121643 33297490 PMC7762337

[B12] Chen C. Chen H. Zhang Y. Thomas H. R. Frank M. H. He Y. . (2020). TBtools: an integrative toolkit developed for interactive analyses of big biological data. Mol. Plant 13, 1194–1202. doi: 10.1016/j.molp.2020.06.009 32585190

[B13] Chen Y. Li W. Turner J. A. Anderson C. T. (2021). PECTATE LYASE LIKE12 patterns the guard cell wall to coordinate turgor pressure and wall mechanics for proper stomatal function in arabidopsis. Plant Cell 33, 3134–3150. doi: 10.1093/plcell/koab163 34109391 PMC8462824

[B14] Chen X. Li C. Wang H. Guo Z. (2019). WRKY transcription factors: evolution, binding, and action. Phytopathol. Res. 1, 1–15. doi: 10.1186/s42483-019-0022-x

[B15] Chinnusamy V. Dalal M. Zhu J. K. (2013). “Epigenetic regulation of abiotic stress responses in plants,” in Plant abiotic stress. Eds. Jenks M. A. Hasegawa P. M. (John Wiley & Sons, Inc), 203–229. doi: 10.1002/9781118764374.ch8

[B16] Curtis T. Y. Bo V. Tucker A. Halford N. G. (2018). Construction of a network describing asparagine metabolism in plants and its application to the identification of genes affecting asparagine metabolism in wheat under drought and nutritional stress. Food Energy Secur. 7, e00126. doi: 10.1002/fes3.126 29938110 PMC5993343

[B17] Deng Y. Wang C. Wang N. Wei L. Li W. Yao Y. . (2019). Roles of small-molecule compounds in plant adventitious root development. Biomolecules 9, 420. doi: 10.3390/biom9090420 31466349 PMC6770160

[B18] Dossa K. Wei X. Li D. Fonceka D. Zhang Y. Wang L. (2016). Insight into the AP2/ERF transcription factor superfamily in sesame and expression profiling of DREB subfamily under drought stress. BMC Plant Biol. 16, 1–16. doi: 10.1186/s12870-016-0859-4 27475988 PMC4967514

[B19] Dreyer I. Sussmilch F. C. Fukushima K. Riadi G. Becker D. Schultz J. . (2021). How to grow a tree: plant voltage-dependent cation channels in the spotlight of evolution. Trends Plant Sci. 26, 41–52. doi: 10.1016/j.tplants.2020.07.011 32868178

[B20] Drummond A. J. Suchard M. A. Xie D. Rambaut A. (2012). Bayesian Phylogenetics with BEAUti and the BEAST 1.7. Mol. Biol. Evol. 29, 1969–1973. doi: 10.1093/molbev/mss075 22367748 PMC3408070

[B21] Eulgem T. Somssich I. E. (2007). Networks of WRKY transcription factors in defense signaling. Curr. Opin. Plant Biol. 10, 366–371. doi: 10.1016/j.pbi.2007.04.020 17644023

[B22] Frederickson Matika D. E. Loake G. J. (2014). Redox regulation in plant immune function. Antioxid Redox Signal 21, 1373–1388. doi: 10.1089/ars.2013.5679 24206122 PMC4158969

[B23] Frey K. Pucker B. (2020). Animal, fungi, and plant genome sequences harbor different non-canonical splice sites. Cells 9, 458. doi: 10.3390/cells9020458 32085510 PMC7072748

[B24] Galván-Gordillo S. V. Martínez-Navarro A. C. Xoconostle-Cázares B. Ruiz-Medrano R. (2016). Bioinformatic analysis of arabidopsis reverse transcriptases with a zinc-finger domain. Biologia 71, 1223–1229. doi: 10.1515/biolog-2016-0145

[B25] Ge M. Liu Y. Jiang L. Wang Y. Lv Y. Zhou L. . (2018). Genome-wide analysis of maize NLP transcription factor family revealed the roles in nitrogen response. Plant Growth Regul. 84, 95–105. doi: 10.1007/s10725-017-0324-x

[B26] Ghorbani R. Zakipour Z. Alemzadeh A. Razi H. (2020). Genome-wide analysis of AP2/ERF transcription factors family in *Brassica napus* . Physiol. Mol. Bio.l Plants 26, 1463–1476. doi: 10.1007/s12298-020-00832-z PMC7326749 32647461

[B27] Glenfield C. Innan H. (2021). Gene duplication and gene fusion are important drivers of tumourigenesis during cancer evolution. Genes 12, 1376. doi: 10.3390/genes12091376 34573358 PMC8466788

[B28] Golomb B. L. Yu A. O. Coates L. C. Marco M. L. (2018). The *Lactococcus lactis* KF 147 nonribosomal peptide synthetase/polyketide synthase system confers resistance to oxidative stress during growth on plant leaf tissue lysate. Microbiologyopen 7, e00531. doi: 10.1002/mbo3.531 28921941 PMC5822349

[B29] He D. Liang R. Long T. Yang Y. Wu C. (2021). Rice RBH1 encoding a pectate lyase is critical for apical panicle development. Plants 10, 271. doi: 10.3390/plants10020271 33573206 PMC7912155

[B30] Hrmova M. Gilliham M. (2018). Plants fighting back: to transport or not to transport, this is a structural question. Curr. Opin. Plant Biol. 46, 68–76. doi: 10.1016/j.pbi.2018.07.006 30138844

[B31] Huang H. Ullah F. Zhou D. X. Yi M. Zhao Y. (2019). Mechanisms of ROS regulation of plant development and stress responses. Front. Plant Sci. 10. doi: 10.3389/fpls.2019.00800 PMC6603150 31293607

[B32] Hu B. Jin J. Guo A. Y. Zhang H. Luo J. Gao G. (2015). GSDS 2.0: an upgraded gene feature visualization server. Bioinformatics 31, 1296–1297. doi: 10.1093/bioinformatics/btu817 25504850 PMC4393523

[B33] Jan R. Khan M. A. Asaf S. Lee I. J. Kim K. M. (2021). Over-expression of chorismate mutase enhances the accumulation of salicylic acid, lignin, and antioxidants in response to the white-backed plant hopper in rice plants. Antioxidants 10, 1680. doi: 10.3390/antiox10111680 34829551 PMC8614942

[B34] Jiao J. Peng D. (2018). Wheat microRNA1023 suppresses invasion of *Fusarium graminearum via* targeting and silencing FGSG_03101. J. Plant Interact. 13, 514–521. doi: 10.1080/17429145.2018.1528512

[B35] Ji X. J. Mao X. Hao Q. T. Liu B. L. Xue J. A. Li R. Z. (2018). Splice variants of the castor WRI1 gene up regulate fatty acid and oil biosynthesis when expressed in tobacco leaves. Int. J. Mol. Sci. 19, 146. doi: 10.3390/ijms19010146 29303957 PMC5796095

[B36] Jin X. (2022). Regulatory network of Serine/Arginine-rich (SR) proteins: the molecular mechanism and physiological function in plants. international journal of molecular sciences. Int. J. Mol. Sci. 23, 10147. doi: 10.3390/ijms231710147 36077545 PMC9456285

[B37] Karlik E. (2021). Why lncRNAs were not conserved? is it for adaptation? Front. Life Sci. RT​​ 2, 103–110. doi: 10.51753/flsrt.1027595

[B38] Karray A. Alonazi M. Jallouli R. Alanazi H. Ben Bacha A. (2022). A proteinaceous alpha-amylase inhibitor from *Moringa oleifera* leaf extract: purification, characterization, and insecticide effects against *C. maculates* insect larvae. Molecules 27, 4222. doi: 10.3390/molecules27134222 35807466 PMC9268253

[B39] Kato Y. Sakamoto W. (2018). FtsH protease in the thylakoid membrane: physiological functions and the regulation of protease activity. Front. Plant Sci. 9. doi: 10.3389/fpls.2018.00855 PMC6019477 29973948

[B40] Kellogg M. K. Miller S. C. Tikhonova E. B. Karamyshev A. L. (2021). SRPassing co-translational targeting: the role of the signal recognition particle in protein targeting and mRNA protection. Int. J. Mol. Sci. 22, 6284. doi: 10.3390/ijms22126284 34208095 PMC8230904

[B41] Kenzior A. Folk W. R. (2015). *Arabidopsis thaliana* MSI4/FVE associates with members of a novel family of plant specific PWWP/RRM domain proteins. Plant Mol. Biol. 87, 329–339. doi: 10.1007/s11103-014-0280-z 25600937

[B42] Khan A. W. Garg V. Roorkiwal M. Golicz A. A. Edwards D. Varshney R. K. (2020). Super-pangenome by integrating the wild side of a species for accelerated crop improvement. Trends Plant Sci. 25, 148–158. doi: 10.1016/j.tplants.2019.10.012 31787539 PMC6988109

[B43] Khanna-Chopra R. (2012). Leaf senescence and abiotic stresses share reactive oxygen species-mediated chloroplast degradation. Protoplasma 249, 469–481. doi: 10.1007/s00709-011-0308-z 21805384

[B44] Kharabian A. (2010). An efficient computational method for screening functional SNPs in plants. J. Theor. Biol. 265, 55–62. doi: 10.1016/j.jtbi.2010.04.017 20406646

[B45] Kumar M. Brar A. Yadav M. Chawade A. Vivekanand V. Pareek N. (2018a). Chitinases–potential candidates for enhanced plant resistance towards fungal pathogens. Agriculture 8, 88. doi: 10.3390/agriculture8070088

[B46] Kumar S. Stecher G. Li M. Knyaz C. Tamura K. (2018b). MEGA X: molecular evolutionary genetics analysis across computing platforms. Mol. Biol. Evol. 35, 1547. doi: 10.1093/molbev/msy096 29722887 PMC5967553

[B47] Laloum T. Martín G. Duque P. (2018). Alternative splicing control of abiotic stress responses. Trends Plant Sci. 23, 140–150. doi: 10.1016/j.tplants.2017.09.019 29074233

[B48] Lanciano S. Mirouze M. (2018). Transposable elements: all mobile, all different, some stress responsive, some adaptive? Curr. Opin. Genet. Dev. 49, 106–114. doi: 10.1016/j.gde.2018.04.002 29705597

[B49] Letunic I. Bork P. (2018). 20 years of the SMART protein domain annotation resource. Nucleic Acids Res. 46, D493–D496. doi: 10.1093/nar/gkx922 29040681 PMC5753352

[B50] Li W. Guan Q. Wang Z. Y. Wang Y. Zhu J. (2013). A bi-functional xyloglucan galactosyltransferase is an indispensable salt stress tolerance determinant in arabidopsis. Mol. Plant 6, 1344–1354. doi: 10.1093/mp/sst062 23571490

[B51] Li D. He Y. Li S. Shi S. Li L. Liu Y. . (2021). Genome-wide characterization and expression analysis of AP2/ERF genes in eggplant (*Solanum melongena* l.). Plant Physiol. Biochem. 167, 492–503. doi: 10.1016/j.plaphy.2021.08.006 34425394

[B52] Li D. Liu P. Yu J. Wang L. Dossa K. Zhang Y. . (2017). Genome-wide analysis of WRKY gene family in the sesame genome and identification of the WRKY genes involved in responses to abiotic stresses. BMC Plant Biol. 17, 1–19. doi: 10.1186/s12870-017-1099-y 28893196 PMC5594535

[B53] Li X. Tao S. Wei S. Ming M. Huang X. Zhang S. . (2018). The mining and evolutionary investigation of AP2/ERF genes in pear (Pyrus). BMC Plant Biol. 18, 46. doi: 10.1186/s12870-018-1265-x 29558898 PMC5859658

[B54] Liu C. Fukumoto T. Matsumoto T. Gena P. Frascaria D. Kaneko T. . (2013). Aquaporin OsPIP1; 1 promotes rice salt resistance and seed germination. Plant Physiol. Biochem. 63, 151–158. doi: 10.1016/j.plaphy.2012.11.018 23262183

[B55] Liu A. Liu C. Lei H. Wang Z. Zhang M. Yan X. . (2020). Phylogenetic analysis and transcriptional profiling of WRKY genes in sunflower (*Helianthus annuus* l.): Genetic diversity and their responses to different biotic and abiotic stresses. Ind. Crops Prod. 148, 112268. doi: 10.1016/j.indcrop.2020.112268

[B56] Lüdke D. Roth C. Kamrad S. A. Messerschmidt J. Hartken D. Appel J. . (2021). Functional requirement of the arabidopsis importin-α nuclear transport receptor family in autoimmunity mediated by the NLR protein SNC1. Plant J. 105, 994–1009. doi: 10.1111/tpj.15082 33210758

[B57] Lu C. Liu H. Jiang D. Wang L. Jiang Y. Tang S. . (2019). *Paecilomyces variotii* extracts (ZNC) enhance plant immunity and promote plant growth. Plant Soil 441, 383–397. doi: 10.1007/s11104-019-04130-w

[B58] Luo M. Cheng K. Xu Y. Yang S. Wu K. (2017 2147). Plant responses to abiotic stress regulated by histone deacetylases. Front. Plant Sci. 8. doi: 10.3389/fpls.2017.02147 PMC5737090 29326743

[B59] Ma W. Kong Q. Arondel V. Kilaru A. Bates P. D. Thrower N. A. . (2013). Wrinkled1, a ubiquitous regulator in oil accumulating tissues from arabidopsis embryos to oil palm mesocarp. PloS One 8, e68887. doi: 10.1371/journal.pone.0068887 23922666 PMC3724841

[B60] Ma L. Li G. (2021). Arabidopsis FAR-RED ELONGATED HYPOCOTYL3 negatively regulates carbon starvation responses. Plant Cell Environ. 44, 1816–1829. doi: 10.1111/pce.14044 33715163

[B61] Mano F. Aoyanagi T. Kozaki A. (2019). Atypical splicing accompanied by skipping conserved micro-exons produces unique WRINKLED1, an AP2 domain transcription factor in rice plants. Plants 8, 207. doi: 10.3390/plants8070207 31277505 PMC6681275

[B62] Marchler-Bauer A. Bo Y. Han L. He J. Lanczycki C. J. Lu S. . (2017). CDD/SPARCLE: functional classification of proteins *via* subfamily domain architectures. Nucleic Acids Res. 45, D200–D203. doi: 10.1093/nar/gkw1129 27899674 PMC5210587

[B63] Martín G. Márquez Y. Mantica F. Duque P. Irimia M. (2021). Alternative splicing landscapes in *Arabidopsis thaliana* across tissues and stress conditions highlight major functional differences with animals. Genome Biol. 22, 35. doi: 10.1186/s13059-020-02258-y 33446251 PMC7807721

[B64] Mindrebo J. T. Nartey C. M. Seto Y. Burkart M. D. Noel J. P. (2016). Unveiling the functional diversity of the alpha/beta hydrolase superfamily in the plant kingdom. Curr. Opin. Struct. Biol. 41, 233–246. doi: 10.1016/j.sbi.2016.08.005 27662376 PMC5687975

[B65] Ming Q. Wang K. Wang J. Liu J. Li X. Wei P. . (2022). The combination of RNA-seq transcriptomics and data-independent acquisition proteomics reveals the mechanisms underlying enhanced salt tolerance by the ZmPDI gene in *Zoysia matrella* [L.] merr. Front. Plant Sci. 13. doi: 10.3389/fpls.2022.970651 PMC9393727 36003810

[B66] Mishra P. Singh A. Roy S. (2022). “Plasma membrane h+-ATPase in plants,” in Cation transporters in plants (Academic Press), 357–373. doi: 10.1016/B978-0-323-85790-1.00012-9

[B67] Moghaddam S. M. Oladzad A. Koh C. Ramsay L. Hart J. P. Mamidi S. . (2021). The tepary bean genome provides insight into evolution and domestication under heat stress. Nat. Commun. 12, 1–14. doi: 10.1038/s41467-021-22858-x 33976152 PMC8113540

[B68] Monné M. Vozza A. Lasorsa F. M. Porcelli V. Palmieri F. (2019). Mitochondrial carriers for aspartate, glutamate and other amino acids: A review. Int. J. Mol. Sci. 20, 4456. doi: 10.3390/ijms20184456 31510000 PMC6769469

[B69] Mu X. Luo J. (2019). Evolutionary analyses of NIN-like proteins in plants and their roles in nitrate signaling. Cell. Mol. Life Sci. 76, 3753–3764. doi: 10.1007/s00018-019-03164-8 31161283 PMC11105697

[B70] Musavizadeh Z. Najafi-Zarrini H. Kazemitabar S. K. Hashemi S. H. Faraji S. Barcaccia G. . (2021). Genome-wide analysis of potassium channel genes in rice: expression of the OsAKT and OsKAT genes under salt stress. Genes 12, 784. doi: 10.3390/genes12050784 34065373 PMC8160896

[B71] Nixon P. J. Michoux F. Yu J. Boehm M. Komenda J. (2010). Recent advances in understanding the assembly and repair of photosystem II. Ann. Bot. 106, 1–16. doi: 10.1093/aob/mcq059 20338950 PMC2889791

[B72] Okada K. Fujiwara S. Tsuzuki M. (2020). Energy conservation in photosynthetic microorganisms. J. Gen. Appl. Microbiol. 66, 59–65. doi: 10.2323/jgam.2020.02.002 32336724

[B73] Olmedo G. Guzmán P. (2008). Processing precursors with RNase III in plants. Plant Sci. 175, 741–746. doi: 10.1016/j.plantsci.2008.07.001

[B74] Ono E. Waki T. Oikawa D. Murata J. Shiraishi A. Toyonaga H. . (2020). Glycoside-specific glycosyltransferases catalyze regio-selective sequential glucosylations for a sesame lignan, sesaminol triglucoside. Plant J. 101, 1221–1233. doi: 10.1111/tpj.14586 31654577

[B75] Pandey S. Prasad A. Sharma N. Prasad M. (2020). Linking the plant stress responses with RNA helicases. Plant Sci. 299, 110607. doi: 10.1016/j.plantsci.2020.110607 32900445

[B76] Pandey P. Ramegowda V. Senthil-Kumar M. (2015). Shared and unique responses of plants to multiple individual stresses and stress combinations: physiological and molecular mechanisms. Front. Plant Sci. 6. doi: 10.3389/fpls.2015.00723 PMC4584981 26442037

[B77] Parakkunnel R. Bhojaraja Naik K. Susmita C. Girimalla V. Bhaskar K. U. Sripathy K. V. . (2022). Evolution and co-evolution: insights into the divergence of plant heat shock factor genes. Physiol. Mol. Biol. Plants 28, 1029–1047. doi: 10.1007/s12298-022-01183-7 35722513 PMC9203654

[B78] Parakkunnel R. Bindhani N. Purru S. Lakhanpaul S. Venkataramanna Bhat K. (2020). Adaptive evolution and response to phytoplasma: A genome-wide study of TCP transcription factors in *Sesamum indicum* l. Ann. Appl. Biol. 176, 75–95. doi: 10.1111/aab.12561

[B79] Pasek S. Risler J. L. Brézellec P. (2006). Gene fusion/fission is a major contributor to evolution of multi-domain bacterial proteins. Bioinformatics 22, 1418–1423. doi: 10.1093/bioinformatics/btl135 16601004

[B80] Poveda J. (2020). *Trichoderma parareesei* favors the tolerance of rapeseed (*Brassica napus* l.) to salinity and drought due to a chorismate mutase. Agronomy 10, 118. doi: 10.3390/agronomy10010118

[B81] Pu L. Cheng L. Li A. Liang S. Wei Q. Wu S. . (2022). Effects of clonal integration on allelopathy of invasive plant *Wedelia trilobata* under heterogeneous light conditions. J. Plant Ecol. 15, 663–671. doi: 10.1093/jpe/rtab028

[B82] Qin Y. Yu H. Cheng S. Liu Z. Yu C. Zhang X. . (2022). Genome-wide analysis of the WRKY gene family in *Malus domestica* and the role of MdWRKY70L in response to drought and salt stresses. Genes 13, 1068. doi: 10.3390/genes13061068 35741830 PMC9222762

[B83] Raikwar S. Srivastava V. K. Gill S. S. Tuteja R. Tuteja N. (2015). Emerging importance of helicases in plant stress tolerance: characterization of *Oryza sativa* repair helicase XPB2 promoter and its functional validation in tobacco under multiple stresses. Front. Plant Sci. 6. doi: 10.3389/fpls.2015.01094 PMC4679908 26734018

[B84] Rambaut A. Drummond A. J. Xie D. Baele G. Suchard M. A. (2018). Posterior summarization in Bayesian phylogenetics using tracer 1.7. Syst. Biol. 67, 901–904. doi: 10.1093/sysbio/syy032 29718447 PMC6101584

[B85] Rawat N. Singla-Pareek S. L. Pareek A. (2021). Membrane dynamics during individual and combined abiotic stresses in plants and tools to study the same. Physiol. Plant 171, 653–676. doi: 10.1111/ppl.13217 32949408

[B86] Ren H. Su Q. Hussain J. Tang S. Song W. Sun Y. . (2021). Slow anion channel GhSLAC1 is essential for stomatal closure in response to drought stress in cotton. J. Plant Physiol. 258, 153360. doi: 10.1016/j.jplph.2020.153360 33482420

[B87] Riaz M. W. Lu J. Shah L. Yang L. Chen C. Mei X. D. . (2021). Expansion and molecular characterization of AP2/ERF gene family in wheat (*Triticum aestivum* l.). Front. Genet. 12. doi: 10.3389/fgene.2021.63215 PMC8044323 33868370

[B88] Rona G. B. Eleutherio E. C. Pinheiro A. S. (2016). PWWP domains and their modes of sensing DNA and histone methylated lysines. Biophys. Rev. 8, 63–74. doi: 10.1007/s12551-015-0190-6 28510146 PMC5425739

[B89] Shi Y. Phan H. Liu Y. Cao S. Zhang Z. Chu C. . (2020). Glycosyltransferase OsUGT90A1 helps protect the plasma membrane during chilling stress in rice. J. Exp. Bot. 71, 2723–2739. doi: 10.1093/jxb/eraa025 31974553 PMC7210772

[B90] Shopan J. Mou H. Zhang L. Zhang C. Ma W. Walsh J. A. . (2017). Eukaryotic translation initiation factor 2B-beta (eIF 2Bβ), a new class of plant virus resistance gene. Plant J. 90, 929–940. doi: 10.1111/tpj.13519 28244149

[B91] Shu K. Yang W. (2017). E3 ubiquitin ligases: ubiquitous actors in plant development and abiotic stress responses. J. Plant Physiol. 58, 1461–1476. doi: 10.1093/pcp/pcx071 PMC5914405 28541504

[B92] Song Q. Bari A. Li H. Chen L. L. (2020). Identification and analysis of micro-exons in AP2/ERF and MADS gene families. FEBS Open Bio 10, 2564–2577. doi: 10.1002/2211-5463.12990 PMC7714060 32986930

[B93] Stankovic N. Schloesser M. Joris M. Sauvage E. Hanikenne M. Motte P. (2016). Dynamic distribution and interaction of the arabidopsis SRSF1 subfamily splicing factors. Plant Physiol. 170, 1000–1013. doi: 10.1104/pp.15.01338 26697894 PMC4734559

[B94] Stein O. Granot D. (2018). Plant fructokinases: evolutionary, developmental, and metabolic aspects in sink tissues. Front. Plant Sci. 9. doi: 10.3389/fpls.2018.00339 PMC5864856 29616058

[B95] Suh A. (2021). “Horizontal transfer of transposons as genomic fossils of host-parasite interactions,” in The evolution and fossil record of parasitism (Springer, Cham), 451–463.

[B96] Su T. Li X. Yang M. Shao Q. Zhao Y. Ma C. . (2020). Autophagy: an intracellular degradation pathway regulating plant survival and stress response. Front. Plant Sci. 11. doi: 10.3389/fpls.2020.00164 PMC7058704 32184795

[B97] Su Y. Ma Z. Mao J. Li W. Cao X. Chen B. (2022). Genome-wide identification and characterization of the strawberry (*Fragaria vesca*) FvAP2/ERF gene family in abiotic stress. Plant Mol. Biol. Rep. 40, 646–660. doi: 10.1007/s11105-022-01343-9

[B98] Sun L. R. Yue C. M. Hao F. S. (2019). Update on roles of nitric oxide in regulating stomatal closure. Plant Signal. Behav. 14, e1649569. doi: 10.1080/15592324.2019.1649569 31370725 PMC6768244

[B99] Tao Y. Luo H. Xu J. Cruickshank A. Zhao X. Teng F. . (2021). Extensive variation within the pan-genome of cultivated and wild sorghum. Nat. Plants 7, 766–773. doi: 10.1038/s41477-021-00925-x 34017083

[B100] Theriault G. Nkongolo K. K. (2017). Evidence of prokaryote like protein associated with nickel resistance in higher plants: horizontal transfer of TonB-dependent receptor/protein in betula genus or *de novo* mechanisms? Heredity 118, 358–365. doi: 10.1111/tpj.15486 27804963 PMC5345603

[B101] Tian P. Lin Z. Lin D. Dong S. Huang J. Huang T. . (2021). The pattern of DNA methylation alteration, and its association with the changes of gene expression and alternative splicing during phosphate starvation in tomato. Plant J. 108, 841–858. doi: 10.1111/tpj.15486 34492142

[B102] Tischkau S. A. (2020). Mechanisms of circadian clock interactions with aryl hydrocarbon receptor signalling. Eur. J. Neurosci. 51, 379–395. doi: 10.1111/ejn.14361 30706546 PMC9530885

[B103] Tranchant-Dubreuil C. Rouard M. Sabot F. (2019). Plant pangenome: impacts on phenotypes and evolution. Annu. Plant Rev. 2, 453–78. doi: 10.1002/9781119312994.apr0664

[B104] Uluisik S. Seymour G. B. (2020). Pectate lyases: Their role in plants and importance in fruit ripening. Food Chem. 309, 125559. doi: 10.1016/j.foodchem.2019.125559 31679850

[B105] Upadhyaya D. C. Bagri D. S. Upadhyaya C. P. Kumar A. Thiruvengadam M. Jain S. K. (2021). Genetic engineering of potato (*Solanum tuberosum* l.) for enhanced α-tocopherols and abiotic stress tolerance. Physiol. Plant 173, 116–128. doi: 10.1111/ppl.13252 33099781

[B106] Vogt J. H. Schippers J. H. (2015). Setting the PAS, the role of circadian PAS domain proteins during environmental adaptation in plants. Front. Plant Sci. 6. doi: 10.3389/fpls.2015.00513 PMC4496561 26217364

[B107] Wang J. Gao S. Peng X. Wu K. Yang S. (2019). Roles of the INO80 and SWR1 chromatin remodeling complexes in plants. Int. J. Mol. Sci. 20, 4591. doi: 10.3390/ijms20184591 31533258 PMC6770637

[B108] Wang L. Zhu J. Li X. Wang S. Wu J. (2018). Salt and drought stress and ABA responses related to bZIP genes from *V. radiata* and *V. angularis* . Gene 651, 152–160. doi: 10.1016/j.gene.2018.02.005 29425824

[B109] Worthington M. Ebina M. Yamanaka N. Heffelfinger C. Quintero C. Zapata Y. P. . (2019). Translocation of a parthenogenesis gene candidate to an alternate carrier chromosome in apomictic *Brachiaria humidicola* . BMC Genom. 20, 41. doi: 10.1186/s12864-018-5392-4 PMC6332668 30642244

[B110] Wu T. Y. Urano D. (2018). Genetic and systematic approaches toward G protein-coupled abiotic stress signaling in plants. Front. Plant Sci. 9. doi: 10.3389/fpls.2018.01378 PMC6158310 30294337

[B111] Xiao W. Chang H. Zhou P. Yuan C. Zhang C. Yao R. . (2015). Genome-wide identification, classification and expression analysis of GHMP genes family in *Arabidopsis thaliana* . Plant Syst. Evol. 301, 2125–2140. doi: 10.1007/s00606-015-1219-9

[B112] Xu W. Tang W. Wang C. Ge L. Sun J. Qi X. . (2020). SiMYB56 confers drought stress tolerance in transgenic rice by regulating lignin biosynthesis and ABA signaling pathway. Front. Plant Sci. 11. doi: 10.3389/fpls.2020.00785 PMC7314972 32625221

[B113] Xu F. Q. Xue H. W. (2019). The ubiquitin-proteasome system in plant responses to environments. Plant Cell Environ. 42, 2931–2944. doi: 10.1111/pce.13633 31364170

[B114] Yamaguchi-Shinozaki K. Shinozaki K. (1994). A novel cis-acting element in an arabidopsis gene is involved in responsiveness to drought, low-temperature, or high-salt stress. Plant Cell 6, 251–264. doi: 10.1105/tpc.6.2.251 8148648 PMC160431

[B115] Yang Y. Liu J. Zhou X. Liu S. Zhuang Y. (2020). Identification of WRKY gene family and characterization of cold stress-responsive WRKY genes in eggplant. PeerJ 8, e8777. doi: 10.7717/peerj.8777 32211240 PMC7083166

[B116] Yang Y. Zhou Y. Chi Y. Fan B. Chen Z. (2017). Characterization of soybean WRKY gene family and identification of soybean WRKY genes that promote resistance to soybean cyst nematode. Sci. Rep. 7, 17804. doi: 10.1038/s41598-017-18235-8 29259331 PMC5736691

[B117] Yu J. Golicz A. A. Lu K. Dossa K. Zhang Y. Chen J. . (2019). Insight into the evolution and functional characteristics of the pan-genome assembly from sesame landraces and modern cultivars. Plant Biotechnol. J. 17, 881–892. doi: 10.1111/pbi.13022 30315621 PMC6587448

[B118] Yu H. Li M. Sandhu J. Sun G. Schnable J. C. Walia H. . (2022). Pervasive misannotation of microexons that are evolutionarily conserved and crucial for gene function in plants. Nat. Commun. 13, 820. doi: 10.1038/s41467-022-28449-8 35145097 PMC8831610

[B119] Yu M. Romer K. A. Nieland T. J. Xu S. Saenz-Vash V. Penman M. . (2011). Exoplasmic cysteine Cys384 of the HDL receptor SR-BI is critical for its sensitivity to a small-molecule inhibitor and normal lipid transport activity. Proc. Natl. Acad. Sci. U.S.A. 108, 12243–12248. doi: 10.1073/pnas.1109078108 21746906 PMC3145699

[B120] Yu G. Xian L. Xue H. Yu W. Rufian J. S. Sang Y. . (2020). A bacterial effector protein prevents MAPK-mediated phosphorylation of SGT1 to suppress plant immunity. PloS Pathog. 16, e1008933. doi: 10.1371/journal.ppat.1008933 32976518 PMC7540872

[B121] Zhang J. De-oliveira-Ceciliato P. Takahashi Y. Schulze S. Dubeaux G. Hauser F. . (2018). Insights into the molecular mechanisms of CO2-mediated regulation of stomatal movements. Curr. Biol. 28, R1356–R1363. doi: 10.1016/j.cub.2018.10.015 30513335

[B122] Zhang N. Yin Y. Liu X. Tong S. Xing J. Zhang Y. . (2017). The E3 ligase TaSAP5 alters drought stress responses by promoting the degradation of DRIP proteins. Plant Physiol. 175, 1878–1892. doi: 10.1104/pp.17.01319 29089392 PMC5717742

[B123] Zheng J. Zhang Z. Tong T. Fang Y. Zhang X. Niu C. . (2021). Genome-wide identification of WRKY gene family and expression analysis under abiotic stress in barley. Agronomy 11, 521. doi: 10.3390/agronomy11030521

[B124] Zhou Y. Lu Q. Zhang J. Zhang S. Weng J. Di H. . (2022a). Genome-wide profiling of splicing and gene fusion during rice black-streaked dwarf virus stress in maize (*Zea mays* l.). Genes 13 (3), 456. doi: 10.3390/genes13030456 35328010 PMC8955601

[B125] Zhou Y. Zhang C. Zhang L. Ye Q. Liu N. Wang M. . (2022b). Gene fusion as an important mechanism to generate new genes in the genus oryza. Genome Biol. 23, 1–23. doi: 10.1186/s13059-022-02696-w 35706016 PMC9199173

[B126] Zhu H. Yang X. Li Q. Guo J. Ma T. Liu S. . (2022). The sweetpotato voltage-gated k+ channel β subunit, KIbB1, positively regulates low-k+ and high-salinity tolerance by maintaining ion homeostasis. Genes 13, 1100. doi: 10.3390/genes13061100 35741862 PMC9222298

