# Supplementary material for: Gene fusions, micro-exons and splice variants define stress signaling by AP2/ERF and WRKY transcription factors in the sesame pan-genome
Source: Front Plant Sci. 2022 Dec 22;13:1076229. doi: 10.3389/fpls.2022.1076229 (PMC9817154; doi:10.3389/fpls.2022.1076229)

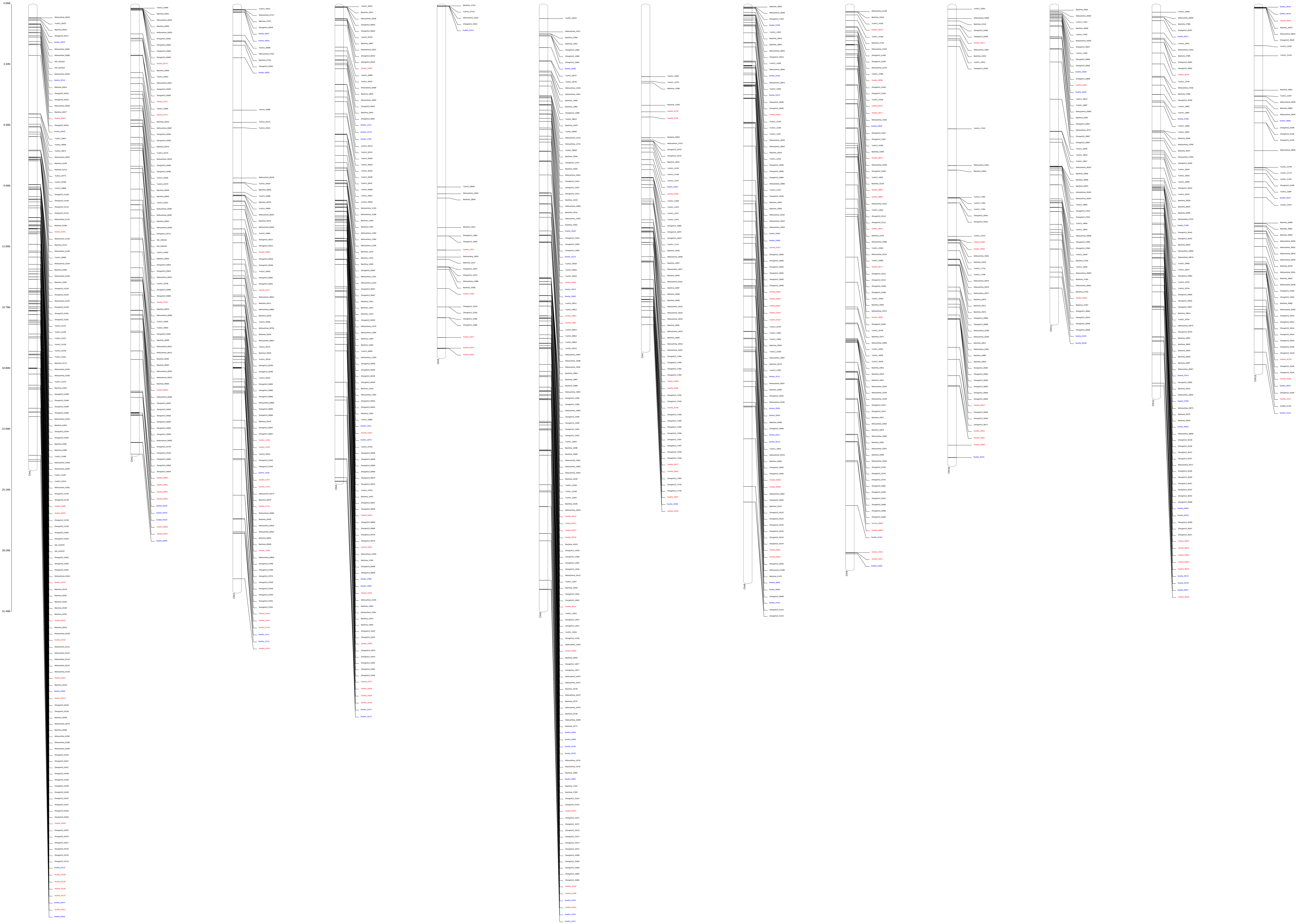

Supplement: Data Sheet 1 — Contains SI-18 (Fig). The AP2/ERF and WRKY genes mapped to the 13 chromosomes of sesame genome. Here the WRKY and AP2/ERF genes from Swetha genome have been represented in ‘blue’ and ‘red’ coloured fonts respectively. Please adjust the zoom to 100% for clear visibility. [file DataSheet_1.pdf]
