# Supplementary material for: Gene fusions, micro-exons and splice variants define stress signaling by AP2/ERF and WRKY transcription factors in the sesame pan-genome
Source: Front Plant Sci. 2022 Dec 22;13:1076229. doi: 10.3389/fpls.2022.1076229 (PMC9817154; doi:10.3389/fpls.2022.1076229)
Supplement: Data Sheet 2 — Contains SI-19 (Fig). The sub-families are coloured as per Swetha AP2/ERF classification given in Fig-2. The colour codes are as following: Blue=DREB; Magenta=ERF; Red=AP2; Green=RAV and Cyan=SOLOIST. Individual Swetha genes are coloured as per the initial classification of Fig-2 to reflect the extent of domain changes in the pan-genome during evolution. [file DataSheet_2.pdf]

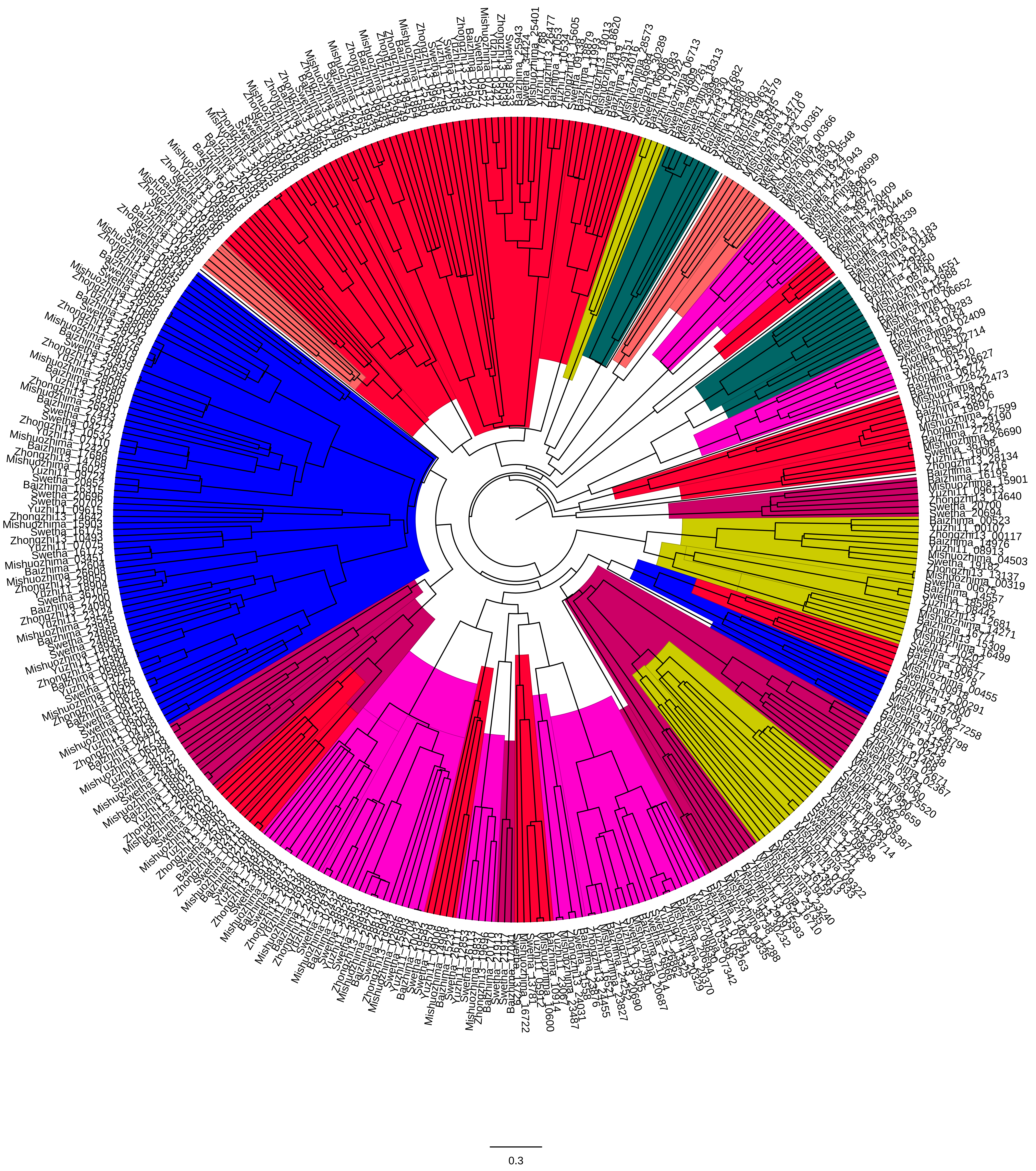
0.3
