# Supplementary material for: Gene fusions, micro-exons and splice variants define stress signaling by AP2/ERF and WRKY transcription factors in the sesame pan-genome
Source: Front Plant Sci. 2022 Dec 22;13:1076229. doi: 10.3389/fpls.2022.1076229 (PMC9817154; doi:10.3389/fpls.2022.1076229)

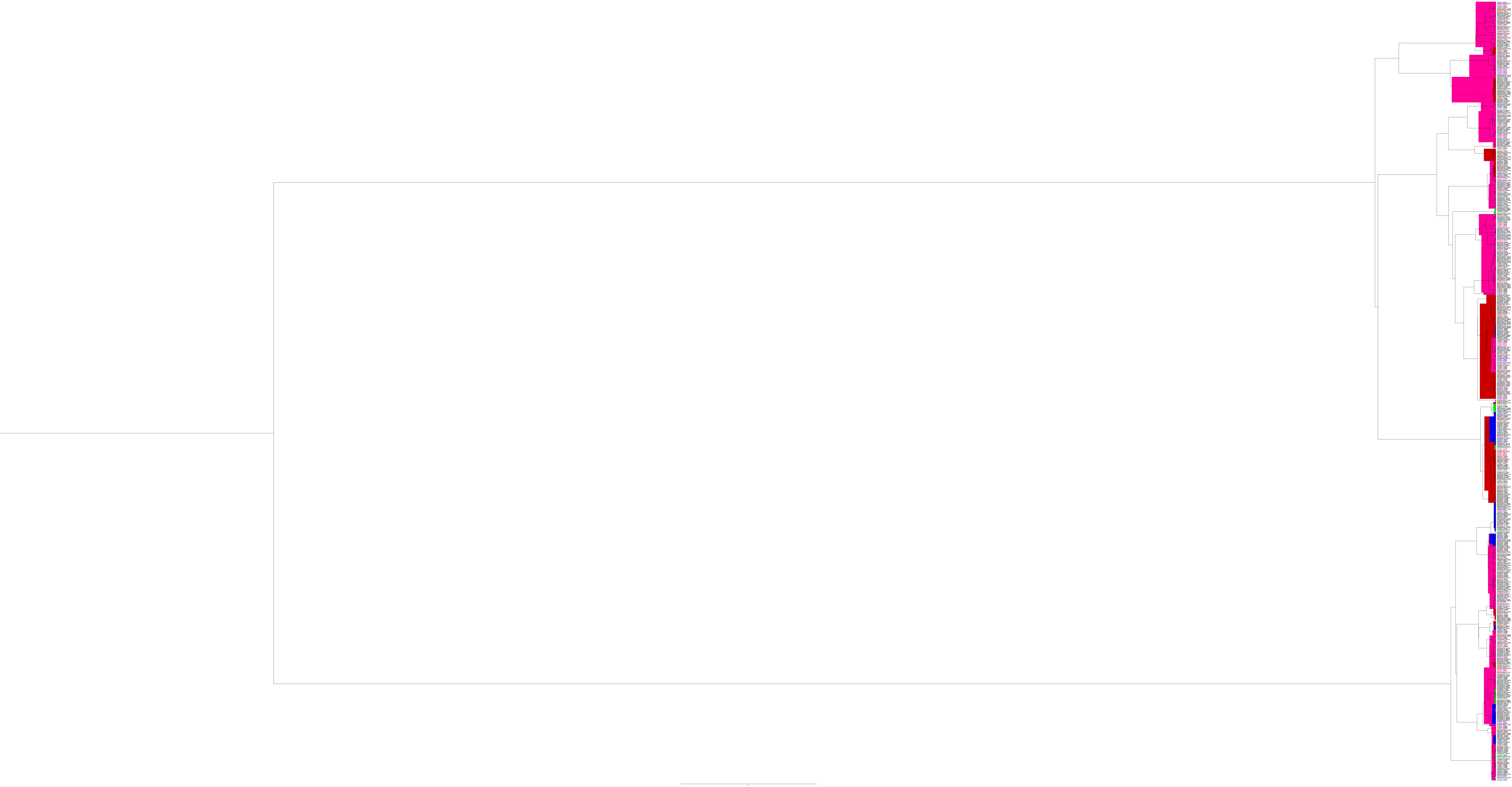

Supplement: Data Sheet 3 — Contains SI-20 (Fig). Different groups are coloured as per classification given in Fig-2. T1=Blue; T2A= Orange; T2B=Magenta, T2C=Red; T2D=Lime yellow; T2E= Purple and T3= Olive. [file DataSheet_3.pdf]
